# Rationale and performances of a data-driven method for computing the duration of pharmacological prescriptions using secondary data sources

**DOI:** 10.1038/s41598-022-10144-9

**Published:** 2022-04-15

**Authors:** Laura Pazzagli, David Liang, Morten Andersen, Marie Linder, Abdul Rauf Khan, Maurizio Sessa

**Affiliations:** 1 Department of Medicine Solna, Centre for Pharmacoepidemiology, Karolinska Institutet, Stockholm, Sweden; 2 Ferring Pharmaceuticals, Copenhagen, Denmark; 3 Department of Drug Design and Pharmacology, University of Copenhagen, Copenhagen, Denmark; 4 Department of Applied Mathematics and Computer Science, Technical University of Denmark, Lyngby, Denmark

**Keywords:** Drug therapy, Epidemiology

## Abstract

The assessment of the duration of pharmacological prescriptions is an important phase in pharmacoepidemiologic studies aiming to investigate persistence, effectiveness or safety of treatments. The Sessa Empirical Estimator (SEE) is a new data-driven method which uses k-means algorithm for computing the duration of pharmacological prescriptions in secondary data sources when this information is missing or incomplete. The SEE was used to compute durations of exposure to pharmacological treatments where simulated and real-world data were used to assess its properties comparing the exposure status extrapolated with the method with the “true” exposure status available in the simulated and real-world data. Finally, the SEE was also compared to a Researcher-Defined Duration (RDD) method. When using simulated data, the SEE showed accuracy of 96% and sensitivity of 96%, while when using real-world data, the method showed sensitivity ranging from 78.0 (nortriptyline) to 95.1% (propafenone). When compared to the RDD, the method had a lower median sensitivity of 2.29% (interquartile range 1.21–4.11%). The SEE showed good properties and may represent a promising tool to assess exposure status when information on treatment duration is not available.

## Introduction

In multiple domains of pharmacoepidemiology, ranging from drug utilization studies to comparative safety and effectiveness research, it is crucial to assess the duration of filled prescriptions of pharmacological treatments (number of days covered by each prescription) to determine exposure status and periods of continuous exposure to medications. The length of exposure to a particular drug during the observational window can be derived from available information on the prescribed dose and daily consumption of the pharmacological treatment for each filled prescription^1–4^. However, not infrequently, such information can be missing in secondary data sources, which usage is exponentially growing in pharmacoepidemiology over the last decades^5,6^.

In this scenario, researchers need to compute the duration of filled prescriptions to determine exposure status and periods of continuous exposure to pharmacological treatments, in a process known as the construction of treatment episodes. Several methods are available for this purpose, including Researcher-Defined Duration (RDD) methods based on assumptions on the daily dose consumption of the treatment, and methods that use temporal distances between prescriptions such as the waiting time distribution (WTD) and its developments^7–11^. Among the RDD methods a common approach uses the Defined Daily Dose (DDD) as assumed daily consumption, a World Health Organization (WHO) measure which has been originally proposed to standardize international comparisons between drug utilization data^12^. Other methods use the assumption of a treatment consumption equal to a fixed daily dose, such as for example 1 tablet per day^13,14^.

This study presents and describes a new data-driven method, the Sessa Empirical Estimator (SEE), for computing the duration of pharmacological prescriptions when information on the prescribed dose and daily consumption is missing or incomplete. The method aims at minimizing rules and assumptions that are commonly used to automate the process of computing prescriptions’ duration. Additionally, this study aims to use simulated and real-world data to evaluate the performances of the method in determining exposure status and the duration of pharmacological prescriptions during the observational window. For context, the method was also compared to a commonly used RDD method with the assumption of a treatment consumption equal to a fixed amount of one unit per day. Finally, this study aims at showing the applicability of the k-means algorithm to the computation of the duration of pharmacological prescriptions, a data-driven method for the individuals’ clustering in the study population.

## Methods

### Simulated data

Simulated data were used to evaluate the performance of correctly computing the duration of filled prescriptions and determine the exposure status of the SEE method. Via simulations, the “true” duration of exposure to a pharmacological treatment and exposure status were generated and then compared with those computed with the SEE method^15^. In a pharmacoepidemiologic setting, it is crucial to simulate data on filled prescriptions that resemble patterns observed in the real world. To mimic the real world, these patterns should include different gradients of re-filling intensity of prescriptions ranging from individuals that refill frequently their medication to those that rarely or never re-fill their prescriptions. Different gradients of re-filling intensity of prescriptions are well captured in summary measures of intensity of medication use in an observational window, e.g. adherence. The simulation was performed by adapting the R functions and procedures described by Allemann and colleagues^16^. One single dataset was simulated containing filled prescription histories of a pharmacological treatment for 1000 patients restricted to an initially filled prescription of 30 days and at least one subsequent filled prescription over an observational window of 2 years. According to the simulation performed by Allemann and colleagues, fixed prescription durations of 1, 2, or 3 months were generated randomly for each subsequent prescription following six adherence trajectories that have been previously observed in real-world data of patients treated with antihypertensive medications (Fig. 2)^17,18^. The six adherence trajectories were used to create six groups mimicking real-world treatment patterns for which adherence was (1) high (95%), (2) medium (50–90%), (3) gradually declining over time, or (4) intermittent (with a change between high and low adherence at regular intervals). Additionally, (5) Partial drop-off (with high adherence initially and partial drop-off after some time) and (6) non-persistence (with one or two refills after the initial fill and no refills afterward) trajectories were simulated^16^. In this simulation, carry-over within and before the observational window was not performed. This approach assumes that all previous filled treatment was consumed before starting the next filled prescription.

### Real-world data

**Figure 1:**
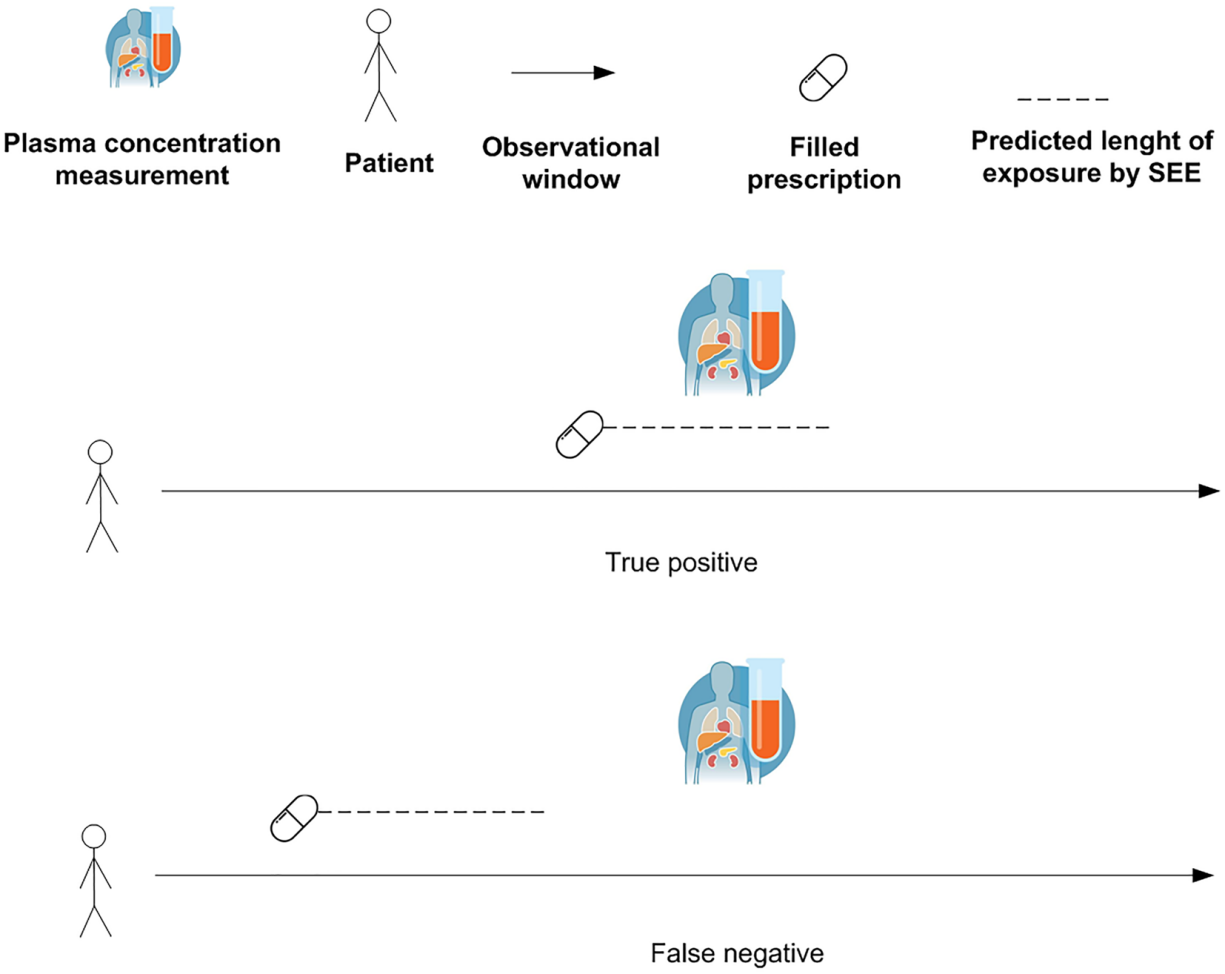
Evaluation of true positives and false negatives in real-world data. SEE = Sessa Empirical Estimator.

The SEE was also tested in real-world data using selected pharmacological treatments with widely used therapeutic drug monitoring in Denmark. Real-world data were retrieved from Danish administrative registers, including the civil registration system (socio-demographic characteristics)^19^, the register for medicinal product statistics (prescription fillings in pharmacies)^20^, the register of causes of death (date/cause of deaths)^21^, the national patient register (hospital admissions/ambulatory visits)^22^, and the register for laboratory results for research (biomarkers and therapeutic drug monitoring results)^23^. These registers have high validity and have previously been used in several pharmacoepidemiologic studies^24^. The linkage between Danish registers is possible since each Danish citizen has a personal identification number which is used as a key of linkage after pseudonymization. The selection included 19 drugs consisting of 11 antiseizure medications (carbamazepine, valproate, phenobarbital, gabapentin, lamotrigine, pregabalin, topiramate, zonisamide, levetiracetam, clobazam, and clonazepam), 5 antidepressant drugs (amitriptyline, citalopram, clomipramine, mirtazapine, and nortriptyline), 2 antiarrhythmic drugs (propafenone and flecainide), and 1 bronchodilator (theophylline). The choice of focusing on these drugs is driven by the analytical strategy in which dates of measurements of plasma concentrations obtained through irregular therapeutic drug monitoring were used to determine the “true” exposure status to pharmacological treatments. In the real-world data, there was no information about the length of filled prescriptions and exposure status in the observational window. Therefore, detectable plasma concentrations of medications were used as a proxy of “true” exposure status. This means that if the patients had a measurable plasma concentration they were considered exposed on that day or relatively close to the date of the plasma measurement (based on the half-life of the drug). To include similar dosing schemes, for antiseizure medications and antidepressants, the analyses were restricted to individuals with a diagnosis of epilepsy (International Classification of Diseases 10th revision, *ICD10* code G40) and depression (ICD10 codes F32 and F33), respectively. Since theophylline, propafenone, and flecainide do not have a different dosing scheme for different indications of use, the preliminary selection of individuals with a pre-specified diagnosis was not made. The study population was composed of individuals that filled their first prescription of the selected drugs and divided into 19 cohorts, one for each drug under investigation. Each individual could contribute to one or more cohorts. The date of the first filled prescription of the drug under investigation was the start of follow-up and will be denoted further in the text as the index date. The study population was followed from the index date to the end of data coverage in 2018. Individuals with detectable plasma concentrations were considered exposed and this was regarded as the “real” exposure status based on the assumption that a patient with a detectable plasma concentration was recently “exposed” to the medication (Fig. 1).

### Sessa empirical estimator (SEE)

The SEE is an algorithm composed of multiple steps aiming to compute the duration of filled prescriptions when information regarding the “true” duration is not available. The method relies on the availability of individual-level information on the date of filling of a medicinal product for computing the duration of filled prescriptions. It assumes that the duration of a filled prescription is associated with the temporal distance between subsequently filled prescriptions as previously described in the WTD method^7,8,11^. Moreover, it is assumed that the amount dispensed is consumed by the patient during the time between two filled prescriptions. The method aims at clustering temporal distances between filled prescriptions into K groups with similar filled prescription patterns and then assigns durations of filled prescriptions within each of the K groups.

The algorithm steps and rationales are described in the following:

1. Among dates of filling of consecutive prescriptions of all patients within the observational window, the SEE computes the Empirical Cumulative Distribution Functions (ECDF) of temporal distances. To avoid including artificially long temporal distances introduced by early- and delayed-discontinuers, the method retains only 80% of the ECDF.
  *Rationale* Artificially long temporal distances can be generated by using the temporal distance between two subsequent filled prescriptions due to, for example, individuals that stop and re-start treatments (stoppers and re-starters), individuals with poor adherence, individuals with missing information on treatments administered during hospitalizations, etc. Stoppers and re-starters are individuals that start medication at one point in time and stop (e.g., due to an adverse reaction to the pharmacological treatment) for a long period before starting the medication again. This problem is solved by cutting the last 20% of the ECDF, which includes the very long temporal distances among consecutive prescriptions.
2. For each individual in the study population, the SEE randomly selects a pair of consecutive filled prescriptions in the observational window.
  *Rationale* By selecting only a random pair of consecutive filled prescriptions, the method removes the overrepresentation of individuals which fill prescriptions of the pharmacological treatment more often due to a lower amount prescribed. If all the prescriptions would be included those individuals would contribute more to the estimation of the median temporal distances than those filling prescriptions less often due to a higher amount prescribed.
3. The temporal distances for the randomly selected filled prescriptions undergo standardization^25^. Subsequently, using the K-means algorithm, the temporal distances are clustered into K groups minimizing the sum of squares from the temporal distances to the assigned cluster centers^26^, and the optimal number of clusters is selected using Silhouette Analysis^27^.
  *Rationale* The temporal distances are standardized by calculating the temporal distances vector’s mean and standard deviation, then each element is scaled by removing the mean and dividing by the standard deviation.
  The standardized vector of temporal distances is used for clustering using the K-means algorithm. Standardization is performed since K-means is a distance-based algorithm that is affected by the scale of a variable^28^. Silhouette Analysis provides the optimal number of clusters measuring the quality of the clustering and it is used to determine how well each object lies within its cluster. A high average silhouette width indicates good clustering. Therefore, the method selects the number of clusters to be used in the K-means clustering algorithm providing the highest value of the average silhouette widths.
4. For each cluster, the SEE builds the probability density function (PDF) of temporal distances to find the median temporal distance. These median values are used as the computed duration of the prescriptions in the cluster.
  *Rationale* This step is performed to obtain the computed durations of filled prescriptions for each cluster (the median temporal distance).
5. Finally, the SEE computes the end of supply for each filled prescription using the computed durations.
  *Rationale* Based on the computed durations of filled prescriptions the end of each supply is computed as the date of filling plus the computed duration.

### Analysis of simulated data

**Figure 2:**
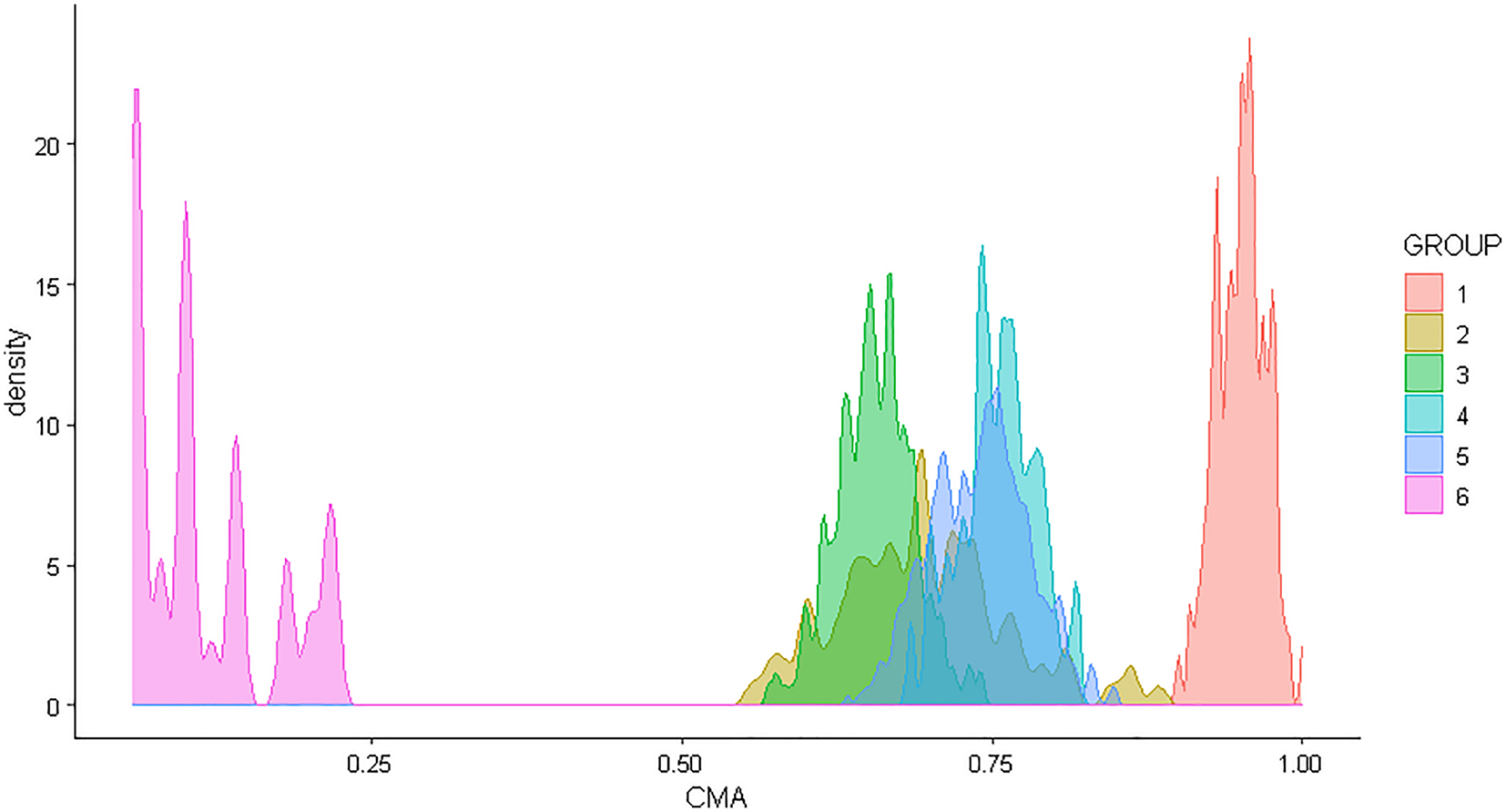
Density plot of adherence for the six groups mimicking real-world treatment patterns for which adherence was 1) high (95%), 2) medium (50–90%), 3) gradually declining over time, or 4) intermittent (with a change between high and low adherence at regular intervals), 5) Partial drop-off (with high adherence initially and partial drop-off after some time), and 6) non-persistence (with one or two refills after the initial fill and no refills afterward) (see also Allemann and colleagues^16^). CMA = Continuous multiple interval measures of medication availability.

For descriptive purposes, a density plot showing the six adherence trajectories simulated is presented (Fig. 2). For each individual in the study population, a random date in the observational window was selected. The “true” versus the SEE assessed exposure status was compared. To evaluate the performances of the method, a confusion matrix^29^ was obtained for the random dates from which accuracy, sensitivity, specificity, positive and negative computed values (PPV/NPV), balanced accuracy, kappa, F1, recall, and precision in assessing exposure status were computed. The classification of individuals in the confusion matrix is reported in Table 1.

**Table 1:** Classification of individuals in the confusion matrix.

Values of the confusion matrix
(1) True positives if at the random date in the observational window individuals were exposed to the pharmacological treatment and the SEE assessed that they were exposed on that specific date
(2) False positives if at the random date in the observational window individuals were not exposed to the pharmacological treatment and the SEE assessed that they were exposed on that specific date
(3) False negatives if at the random date in the observational window individuals were exposed to the pharmacological treatment and the SEE assessed that they were not exposed on that specific date
(4) True negatives if at the random date in the observational window individuals were not exposed to the pharmacological treatment and the SEE assessed that they were not exposed on that specific date

To account for unbalanced groups in the confusion matrix, a sensitivity analysis was performed using a random sample of individuals that were true positives. Finally, to calculate the number of days of misclassification of exposure status, the duration computed by the SEE was compared with the “true” duration of each filled prescription. Moreover, the absolute value of the difference between the “true” duration of each filled prescription versus the duration computed by the SEE was computed separately for true positives, false positives, true negatives, and false negatives.

### Analysis of real-world data

For each cohort, median age and interquartile range (at the index date), the proportion of female sex, and the proportion of individuals filling their first prescription in 1995 (when the register of medicinal product statistics started) were tabulated and presented separately for individuals with and without plasma concentration measurements. Among individuals with measurements of plasma concentration of the drug under investigation, the “true” versus the SEE assessed exposure status was compared and sensitivity was assessed. Individuals were considered truly exposed to the medication at the date of measurement/s if they had a detectable plasma concentration of the medication. To assess if the median duration of different filled prescriptions (e.g., 1^st^, 2^nd^, 3^rd^, 4^th^, etc.) for each cohort was similar during the observational window, boxplots of the temporal distances between consecutive prescriptions were presented. The analysis has been presented separately for individuals with and without measurements to show their comparability in terms of medication use over time. With the final goal of comparing the performances of the SEE, sensitivity was assessed also for an RDD method widely used in the scientific literature which uses the assumption of a treatment consumption equal to the fixed amount of one unit per day (Supplementary Table 1 reports the dosage recommendations in the Danish Summary of Product Characteristic (SmPC) for the 19 drugs under investigation). The comparison between the proposed method and the RDD method has been presented as a bar chart of sensitivity.

### Software used for data analysis

Simulations and data analyses were performed using R 4.0.0 (R Core Team, 2020). Reporting and design of simulations were performed according to relevant guidelines for simulation of data in medical statistics^30^. An R package implementing the SEE is currently available on GitHub (https://github.com/arkh88/SEE_test) and a Shiny app is available online (https://sessaempiricalestimator.shinyapps.io/SessaEmpiricalEstimator/).

## Results

### Simulated data

**Figure 3:**
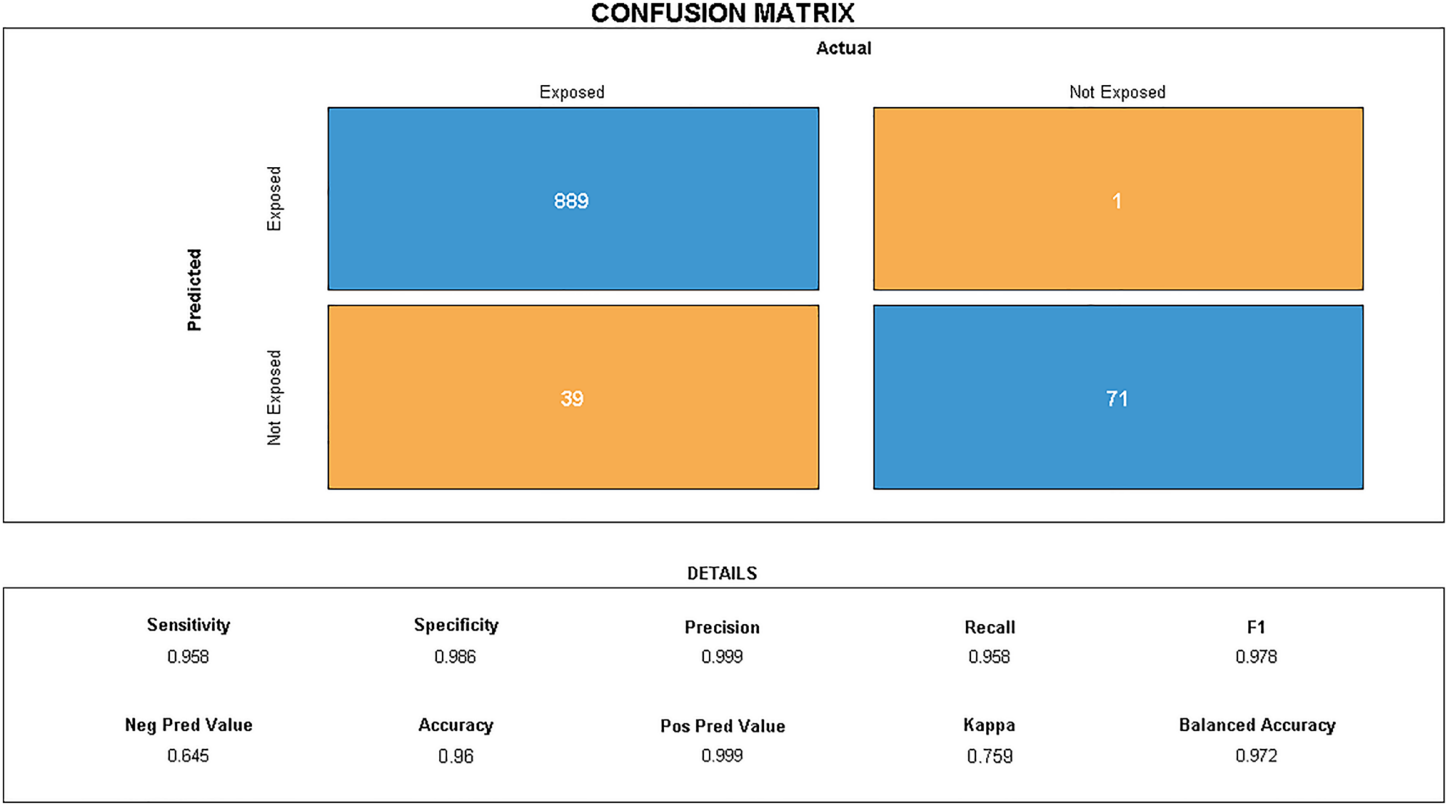
Results of the confusion matrix when comparing “true” versus assessed exposure status (by Sessa Empirical Estimator).

In total, 100 (10%), 93 (9%), 236 (24%), 93 (9%), 379 (38%), and 99 (10%) patients were simulated for groups 1, 2, 3, 4, 5, and 6, respectively, which density of adherence is presented in Fig. 2. The results from the confusion matrix are presented in Fig. 3. The SEE showed an accuracy of 96%*,* sensitivity of 96%, specificity of 99%, precision of 99%, recall of 96%, F1 of 98%, kappa of 76%, NPV of 64%, PPV of 99%, and balanced accuracy of 97% in assessing exposure status. The absolute median number of days of misclassification of exposure was 16 days (interquartile range: 16–44 days) among true positives, true negatives, and false negatives. For false positives, there was one patient with 16 days of misclassification of exposure duration. In the sensitivity analysis using a random sample of individuals that were true positives, similar metrics were obtained except for the NPV for which there was observed an improvement after balancing (Supplementary Fig. 1).

### Real-world data

**Figure 4:**
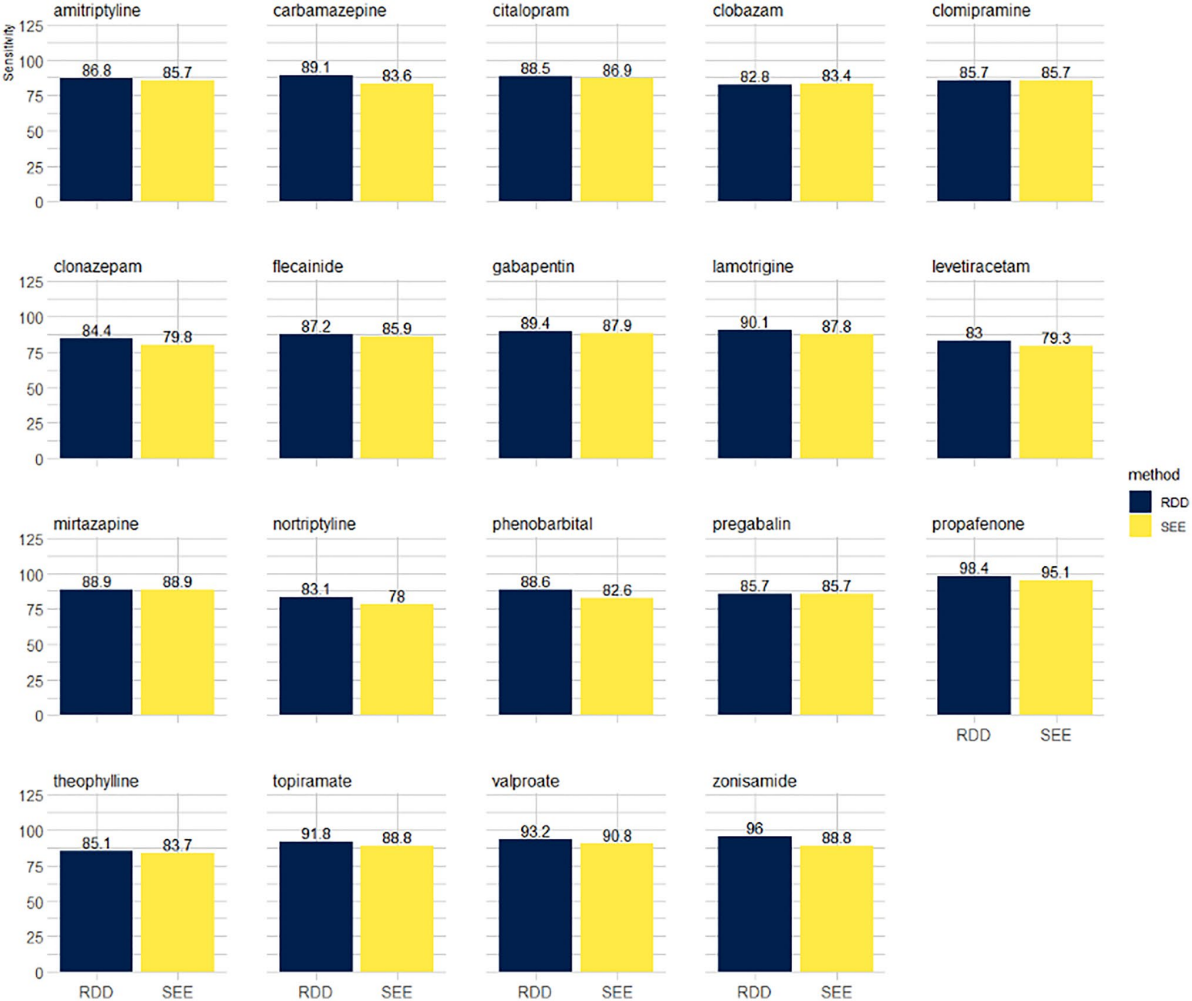
Comparison of the performances (sensitivity) of the Sessa Empirical Estimator (SEE) with the Research-Defined Duration (RDD).

Baseline characteristics of individuals with and without measurements of plasma concentrations in the real-world population are presented in Supplementary Table 2 for all the 19 cohorts. For each drug, the median duration of the temporal distances between consecutive prescriptions is presented in Supplementary Figs. 2–20. Overall, there was comparability of age, sex distribution, and median duration of the temporal distances between consecutive prescriptions among individuals with and without measurements of plasma concentrations. In real-world data, the SEE showed a sensitivity ranging from 78.0% (nortriptyline) to 95.1% (propafenone) in assessing exposure status at the time of measurements of detectable plasma concentrations. When compared to the RDD, the proposed method had a lower median sensitivity of 2.29% (interquartile range 1.21–4.11%) (Fig. 4). The median was computed as the median of the differences in sensitivity between RDD and SEE for all the drugs under investigation.

## Discussion

This study showed good properties of the SEE in terms of accuracy, sensitivity, specificity, NPV, PPV, and balanced accuracy even in presence of a variety of different adherence patterns in our simulated data and good sensitivity in real-world data highlighting several strengths of the method. The approach offers the advantage of computing the duration of a filled prescription when information on the daily dose prescribed is missing or incomplete, minimizing rules and assumptions that are commonly used. We also showed that the proposed method can be used to extend the application of the k-means algorithm to the computation of individuals’ filled prescriptions using temporal distances between filled prescriptions and allowing the data to drive the clustering of individuals in the study population. However, these results should be considered in virtue of a set of limitations. Despite the good properties, in both simulated and real-world data, a quote of misclassification of exposure has been observed. In real-world data, the results should be interpreted with caution since sensitivity alone is not a good proxy for accuracy. Hypothetically, a very long and unrealistic duration of filled prescriptions giving an individual a total adherence of 100%, will give a sensitivity of 100% in the observational window. As we were not able to compare the misclassification in days in the real-world setting, we cannot pose a solid conclusion about the reliability of the SEE in real-world data nor about the comparison of the performances between the method and the RDD. Therefore, it should be emphasized that despite promising, these results are only the cornerstone for future studies in different pharmacoepidemiologic settings to further understand the properties of the proposed method as a new tool for computing the duration of filled prescriptions and better characterize strengths and limitations of the method. The data-driven approach provided by the SEE may be appealing in several domains of pharmacoepidemiology ranging from drug utilization studies to comparative effectiveness research as it can automate the procedures needed for computing the duration of filled prescriptions while keeping good properties. Data-driven assessment of exposure status in secondary data sources is promising in pharmacoepidemiology as it can lead to a population-based assessment of persistence to pharmacological treatments. In turn, such applications may lead to large-scale identifications of individuals with poor or un-optimal persistence as measured by filled prescriptions and understanding of which clinical and socio-demographic characteristics may influence persistence.

## Conclusion

The SEE showed good properties in both simulated and real-world data. The method is a promising tool to assess exposure status when information on treatment duration is not available. However, further investigations are needed to assess the validity of the proposed method in real-world data and different pharmacoepidemiologic settings.

## Supplementary Information

Supplementary Information.

## References

[1] Gardarsdottir H, Souverein PC, Egberts TC, Heerdink ER (2010). Construction of drug treatment episodes from drug-dispensing histories is influenced by the gap length. J. Clin. Epidemiol..

[2] Pazzagli L (2018). Methods for time-varying exposure related problems in pharmacoepidemiology: An overview. Pharmacoepidemiol. Drug Saf..

[3] Meaidi M (2021). Pharmacoepidemiological methods for computing the duration of pharmacological prescriptions using secondary data sources. Eur. J. Clin. Pharmacol..

[4] Pazzagli L (2020). Methods for constructing treatment episodes and impact on exposure-outcome associations. Eur. J. Clin. Pharmacol..

[5] Harpe SE (2009). Using secondary data sources for pharmacoepidemiology and outcomes research. Pharmacother.: The J. Human Pharmacol. Drug Ther..

[6] Soeorg, H., Sverrisdóttir, E., Andersen, M., Lund, T. M. & Sessa, M. The PHARMACOM‐EPI framework for integrating pharmacometric modelling into pharmacoepidemiological research using real‐world data: Application to assess death associated with valproate. *Clin. Pharmacol. Therapeut.* (2021). 10.1002/cpt.2502 34860420

[7] Pottegård A, Hallas J (2013). Assigning exposure duration to single prescriptions by use of the waiting time distribution. Pharmacoepidemiol. Drug Saf..

[8] Stovring H, Pottegard A, Hallas J (2016). Determining prescription durations based on the parametric waiting time distribution. Pharmacoepidemiol. Drug Saf..

[9] Thrane JM, Støvring H, Hellfritzsch M, Hallas J, Pottegård A (2018). Empirical validation of the reverse parametric waiting time distribution and standard methods to estimate prescription durations for warfarin. Pharmacoepidemiol. Drug Saf..

[10] Bødkergaard K (2020). Using the waiting time distribution with random index dates to estimate prescription durations in the presence of seasonal stockpiling. Pharmacoepidemiol. Drug Saf..

[11] Hallas, J., Gaist, D. & Bjerrum, L. The waiting time distribution as a graphical approach to epidemiologic measures of drug utilization. *Epidemiology*, 666–670 (1997). 10.1097/00001648-199710000-00009 9345667

[12] Organization, W. H. Studies in drug utilization: methods and applications. *Studies in drug utilization: methods and applications.* (1979).

[13] Ruokoniemi P (2011). Statin adherence and the risk of major coronary events in patients with diabetes: A nested case–control study. Br. J. Clin. Pharmacol..

[14] Helin-Salmivaara A (2012). Statins and hip fracture prevention–a population based cohort study in women. PLoS ONE.

[15] Boulesteix AL (2020). Introduction to statistical simulations in health research. BMJ Open.

[16] Allemann SS, Dediu D, Dima AL (2019). Beyond adherence thresholds: A simulation study of the optimal classification of longitudinal adherence trajectories from medication refill histories. Front. Pharmacol..

[17] Hargrove JL (2017). Antihypertensive adherence trajectories among older adults in the first year after initiation of therapy. Am. J. Hypertens..

[18] Hansen RA (2009). Adherence: comparison of methods to assess medication adherence and classify nonadherence. Ann. Pharmacother..

[19] Pedersen CB (2011). The Danish civil registration system. Scand. J. Public Health.

[20] Wallach Kildemoes H, Toft Sørensen H, Hallas J (2011). The Danish national prescription registry. Scand. J. Public Health.

[21] Helweg-Larsen K (2011). The Danish register of causes of death. Scand. J. Public Health.

[22] Lynge E, Sandegaard JL, Rebolj M (2011). The Danish national patient register. Scand. J. Public Health.

[23] arendt JFH (2020). existing data sources in clinical epidemiology: laboratory information system databases in Denmark. Clin. Epidemiol..

[24] Nickelsen TN (2001). Data validity and coverage in the Danish National Health Registry. A literature review. Ugeskr. Laeger.

[25] De Amorim RC, Hennig C (2015). Recovering the number of clusters in data sets with noise features using feature rescaling factors. Inf. Sci..

[26] Steinley D (2006). K-means clustering: a half-century synthesis. Br. J. Math. Stat. Psychol..

[27] Zhao S, Sun J, Shimizu K, Kadota K (2018). Silhouette scores for arbitrary defined groups in gene expression data and insights into differential expression results. Biol. Proc. Online.

[28] Cao XH, Stojkovic I, Obradovic Z (2016). A robust data scaling algorithm to improve classification accuracies in biomedical data. BMC Bioinform..

[29] Ting, K. M. in *Encyclopedia of Machine Learning* (eds Claude Sammut & Geoffrey I. Webb) 209–209 (Springer US, 2010).

[30] Burton A, Altman DG, Royston P, Holder RL (2006). The design of simulation studies in medical statistics. Stat. Med..

