# Supplementary material for: Rationale and performances of a data-driven method for computing the duration of pharmacological prescriptions using secondary data sources
Source: Sci Rep. 2022 Apr 15;12:6245. doi: 10.1038/s41598-022-10144-9 (PMC9012860; doi:10.1038/s41598-022-10144-9)
Supplement: Supplementary file 1 — Supplementary Information. [file 41598_2022_10144_MOESM1_ESM.pdf]

Supplementary material

Rationale and performances of a data-driven method for computing the duration of pharmacological prescriptions using secondary data sources

Laura Pazzagli[1*], David Liang[2], Morten Andersen[3], Marie Linder[1], Abdul Rauf Khan[3,4], Maurizio Sessa[3]

[1]Centre for Pharmacoepidemiology, Department of Medicine Solna, Karolinska Institutet, Stockholm, Sweden
[2]Ferring Pharmaceuticals, Copenhagen, Denmark
[3]Department of Drug Design and Pharmacology, University of Copenhagen, Copenhagen, Denmark
[4]Department of Applied Mathematics and Computer Science, Technical University of Denmark, Lyngby, Denmark.

***Corresponding author**
Laura Pazzagli. *ORCID:* https://orcid.org/0000-0002-1908-6073
Centre for Pharmacoepidemiology, Department of Medicine Solna, Karolinska Institutet S-171 76, Stockholm, Sweden.
E-mail: laura.pazzagli@ki.se

Supplementary table 1. Dosage recommendations in the Danish Summary of Product Characteristic (SmPC) for the 19 drugs under investigation.

Drug	Approach	Dosage recommendations in the Danish SmPC
Carbamazepine	2 units per day	**Epilepsy**<br>*Adults and children> 15 years.* Initially 100 mg twice daily Increase by 100 mg every 2 days to maintenance dose 300 mg twice-daily. Further increase to 800-1,200 mg daily may be necessary.
Valproate	2 units per day	**Epilepsy**<br>*Adults.* Initially 600 mg daily as a single dose. The dose is increased to a maintenance dose of 600-1,200 mg daily divided into 1-2 doses.
Phenobarbital	1 unit per day	**Epilepsy**<br>*Adults.* 1-2 mg / kg body weight once daily at bedtime.
Gabapentin	3 units per day	**Epilepsy**<br>*Adults*<br>1st day 300 mg once daily. 2nd day 300 mg twice daily. 3rd day 300 mg 3 times a day.<br>Then step up with 300 mg daily every 2-3 days to a maximum of 3,600 mg daily in 3 divided doses.
Lamotrigine	2 units per day	**Epilepsy**<br>*Adults and children> 13 years*. Initially 25 mg once daily for 2 weeks. Then 50 mg once daily for 2 weeks. Thereafter, the dose is increased by 50-100 mg every one or two weeks for optimal clinical response. Usual maintenance dose 100-300 mg daily in 1 or 2 doses. Some patients need higher doses, eg 500-1,200 mg daily.
Levetiracetam	2 units per day	**Epilepsy**<br>*Adults and children> 16 years.* Initially 250 mg twice daily.<br>After 2 weeks the dose can be increased to 500 mg twice daily. The dose can be increased by a further 250 mg twice daily every 2 weeks to a maximum of 1.5 g twice daily.
Pregabalin	3 units per day	**Epilepsy**<br>*Adults*. Initially 150 mg daily divided into 2-3 doses. The dose can be increased to 300 mg daily after 7 days and to a maximum of 600 mg daily after other 7 days.
Topiramate	2 units per day	**Epilepsy**<br>*Adults*. Initially 25 mg daily in the evening for 1 week. Then the dose is increased at 1-2-week intervals by 25-50 mg per day divided into 2 doses depending on the effect. Usual maintenance dose 200-400 mg daily divided into 2 doses.
Zonisamide	1 unit per day	**Epilepsy**<br>*Adults*. Initially 100 mg once daily.<br>After 2 weeks, the dose can be increased to 200 mg once daily. After a further 2 weeks, the dose may be increased to 300 mg once daily. Usual maintenance dose 300 mg once daily.
Clobazam	2 units per day	**Epilepsy**<br>*Adults*. 10-40 (-80) mg daily in 1-3 doses.

Clonazepam	2 units per day	**Epilepsy**<br>*Adults*. Initially 0.5 mg daily, increase by 0.5 mg every 3 days to a maximum of 6 mg per day in 1-2 doses.
Theophylline	2 units per day	**Asthma or chronic obstructive pulmonary disease**<br>*Adults*. Individual dosage. 10-15 mg / kg body weight per day, usually divided into 2 doses.
Flecainide	2 units per day	**Cardiac arrhythmia**<br>*Older patients.* 100 mg twice daily Dose reduction after 5-6 days.
Propafenone	2 units per day	**Cardiac arrhythmia**<br>*Older patients.* 300 mg daily divided into 2 doses with dose adjustment after 3-4 days. Careful monitoring.
Amitriptyline	2 units per day	**Depression**<br>*Older patients*. Initially 10 mg twice daily, gradually increasing to 100-150 mg daily in 1-2 doses.
Citalopram	1 unit per day	**Depression**<br>*Older patients*. Initially 10 mg daily Maximum 20 mg daily Dose increase should be done slowly under close supervision.
Clomipramine	1 unit per day	**Depression**<br>*Older patients*. Initially 10 mg daily, increasing to normally 30-50 mg daily.
Mirtazapine	1 unit per day	**Depression**<br>*Older patients*. Initially 15-30 mg daily. The usual maintenance dose is 15-45 mg daily. The dose is given once a day at bedtime.
Nortriptyline	2 units per day	**Depression**<br>*Older patients*. Initially 10 mg twice daily, gradually increasing to 75 mg daily in 1-2 doses.

Supplementary table 2. Baseline characteristics of individuals with and without plasma concentration measurements for the 19 cohorts. *N= Number of patients; Plasma concentration measurement (PCM); Interquartile Range (IQR)*

	Age (median – IQR)	% Female sex	% Enrolled in 1995
**Carbamazepine**			
Individuals without PCM (N=29,161)	60.3 (52.3 – 69.0)	49.8	62.5
Individuals with PCM (N=6,923)	51.9 (47.1 – 59.0)	49.3	74.5
**Valproate**			
Individuals without PCM (N=62,009)	70.4 (62.3 – 77.9)	49.9	15.2
Individuals with PCM (N=20,078)	66.7 (59.0 – 77.2)	44.2	13.8
**Phenobarbital**			
Individuals without PCM (N=24,744)	64.4 (56.5 – 72.9)	47.1	38.2
Individuals with PCM (N=2,612)	55.1 (48.7 – 63.5)	44.0	52.2
**Gabapentin**			
Individuals without PCM (N=23,162)	69.0 (62.3 – 75.8)	51.8	0
Individuals with PCM (N=531)	66.9 (53.3 – 70.2)	78.5	0
**Lamotrigine**			
Individuals without PCM (N=59,049)	67.5 (59.1 – 75.5)	50.8	9.2
Individuals with PCM (N=24,471)	64.6 (57.6 – 72.2)	50.2	7.8
**Levetiracetam**			
Individuals without PCM (N=36,886)	69.1 (63.7 – 76.2)	47.6	0
Individuals with PCM (N=24,879)	69.1 (63.5 – 75.3)	49.7	0
**Pregabalin**			
Individuals without PCM (N=7,492)	69.2 (64.1 – 75.5)	59.0	0
Individuals with PCM (N=132)	68.4 (64.8 – 71.1)	77.3	0
**Topiramate**			
Individuals without PCM (N=13,448)	60.6 (54.2 – 67.9)	56.8	0
Individuals with PCM (N=3,053)	57.9 (52.3 – 63.1)	54.3	0
**Zonisamide**			
Individuals without PCM (N=4,360)	65.5 (60.6 – 71.8)	59.2	0
Individuals with PCM (N=2,049)	69.0 (63.9 – 74.1)	56.8	0
**Flecainide**			
Individuals without PCM (N=8,037)	66.9 (61.4 – 72.8)	46.1	19.1
Individuals with PCM (N=206)	65.8 (61.2 – 69.6)	46.1	3.4
**Propafenone**			
Individuals without PCM (N=6,423)	65.4 (56.8 – 69.8)	50.0	11.1
Individuals with PCM (N=64)	68.7 (61.6 – 75.1)	44.6	26.8

**Theophylline**			
Individuals without PCM (N=59,436)	71.4 (64.3 – 77.9)	53.7	52.2
Individuals with PCM (N=156)	62.7 (55.7 – 70.1)	66.7	51.3
**Amitriptyline**			
Individuals without PCM (N=10,503)	71.8 (63.5 – 78.7)	73.2	21.3
Individuals with PCM (N=86)	64.1 (60.0 – 67.4)	61.6	23.3
**Citalopram**			
Individuals without PCM (N=49,922)	75.7 (67.7 – 82.5)	69.0	12.3
Individuals with PCM (N=208)	77.8 (69.6 – 81.8)	79.3	7.2
**Clomipramine**			
Individuals without PCM (N=2,377)	66.3 (59.1 – 73.6)	78.3	43.5
Individuals with PCM (N=46)	68.1 (52.8 – 73.2)	50.0	19.6
**Mirtazapine**			
Individuals without PCM (N=42,753)	78.5 (71.0 – 84.2)	69.5	0
Individuals with PCM (N=98)	80.2 (69.7 – 81.3)	67.3	0
**Nortriptyline**			
Individuals without PCM (N=7,067)	72.3 (64.7 – 79.1)	73.7	17.2
Individuals with PCM (N=663)	71.1 (63.4 – 78.0)	71.2	5.6
**Clobazam**			
Individuals without PCM (N=24,090)	66.1 (59.3 – 72.7)	50.5	8.8
Individuals with PCM (N=2262)	63.4 (55.2 – 68.8)	51.8	10.5
**Clonazepam**			
Individuals without PCM (N=18,895)	62.6 (54.4 – 70.3)	54.0	21.6
Individuals with PCM (N=723)	53.4 (46.6 – 65.5)	62.8	42.5

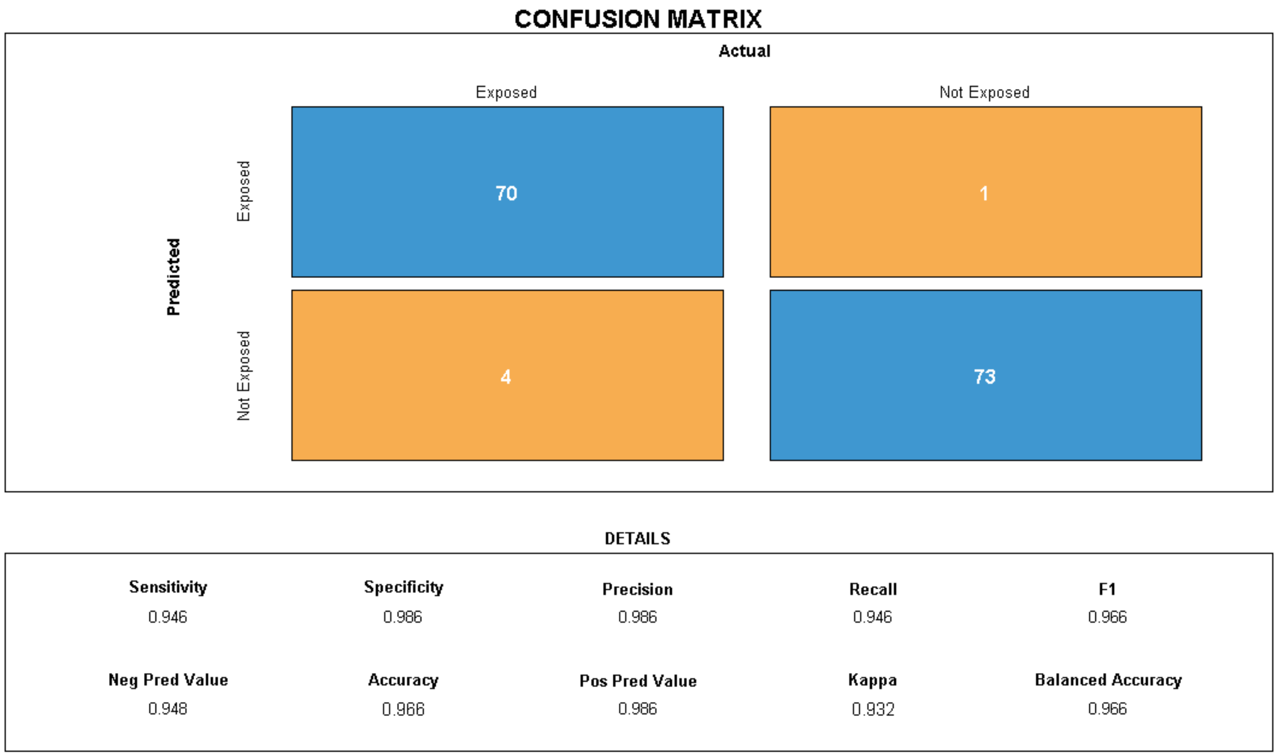

Supplementary figure 1. Results of the confusion matrix when comparing “true” versus assessed exposure status (by Sessa Empirical Estimator) after balancing “true” positives.

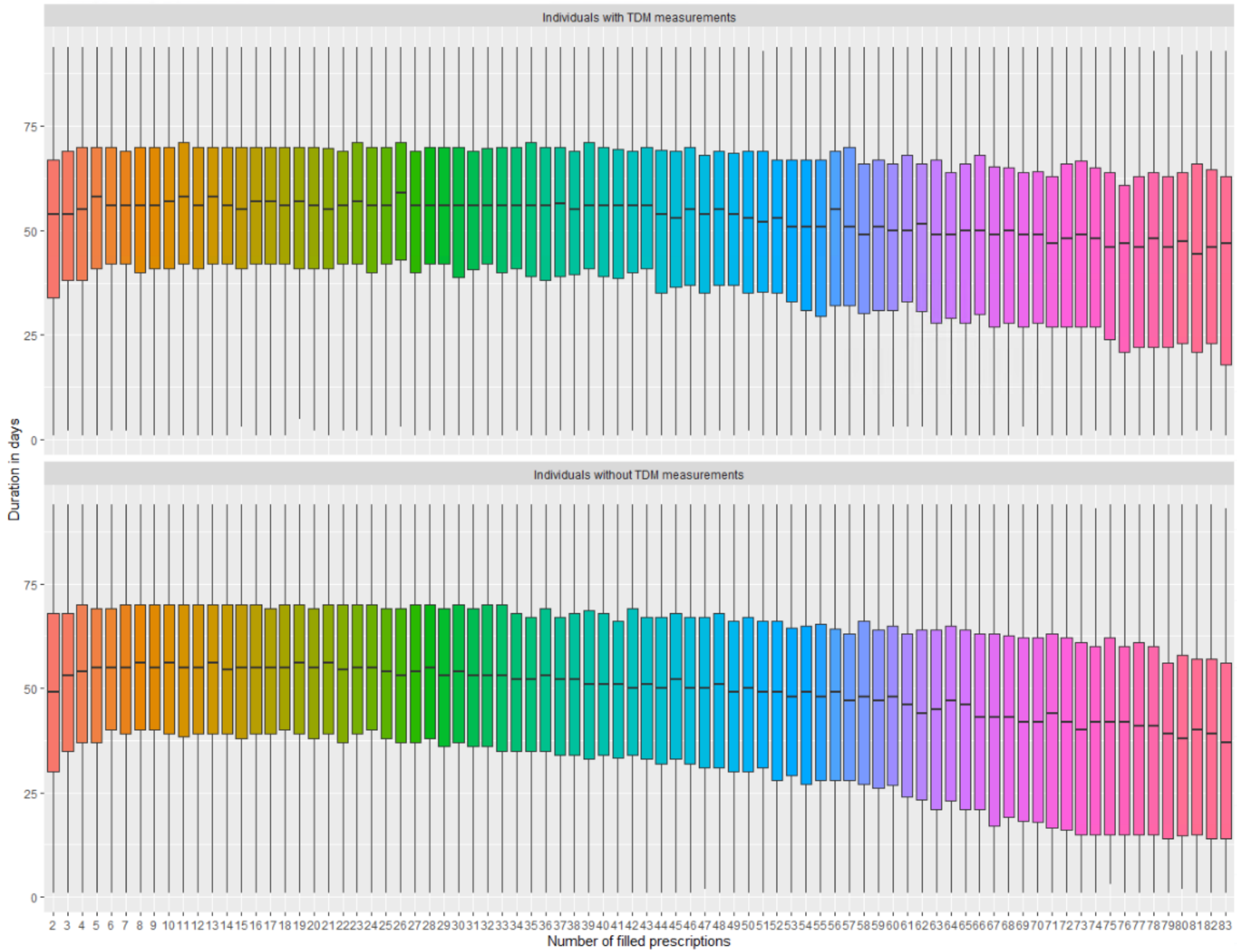

Supplementary figure 2. Median duration of the temporal distances between consecutive prescriptions for carbamazepine. *TDM = therapeutic drug monitoring*.

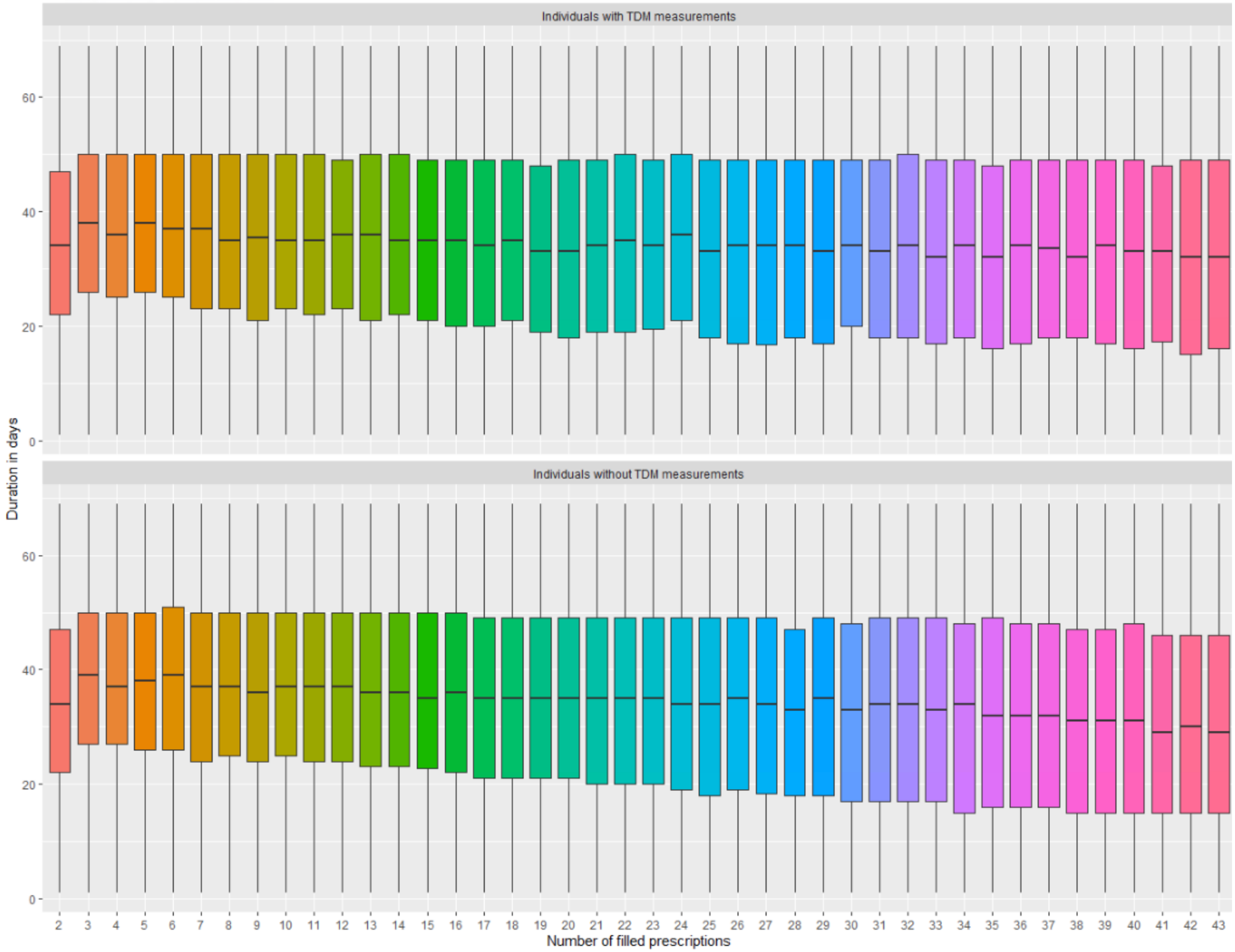

Supplementary figure 3. Median duration of the temporal distances between consecutive prescriptions for valproate. *TDM = therapeutic drug monitoring*.

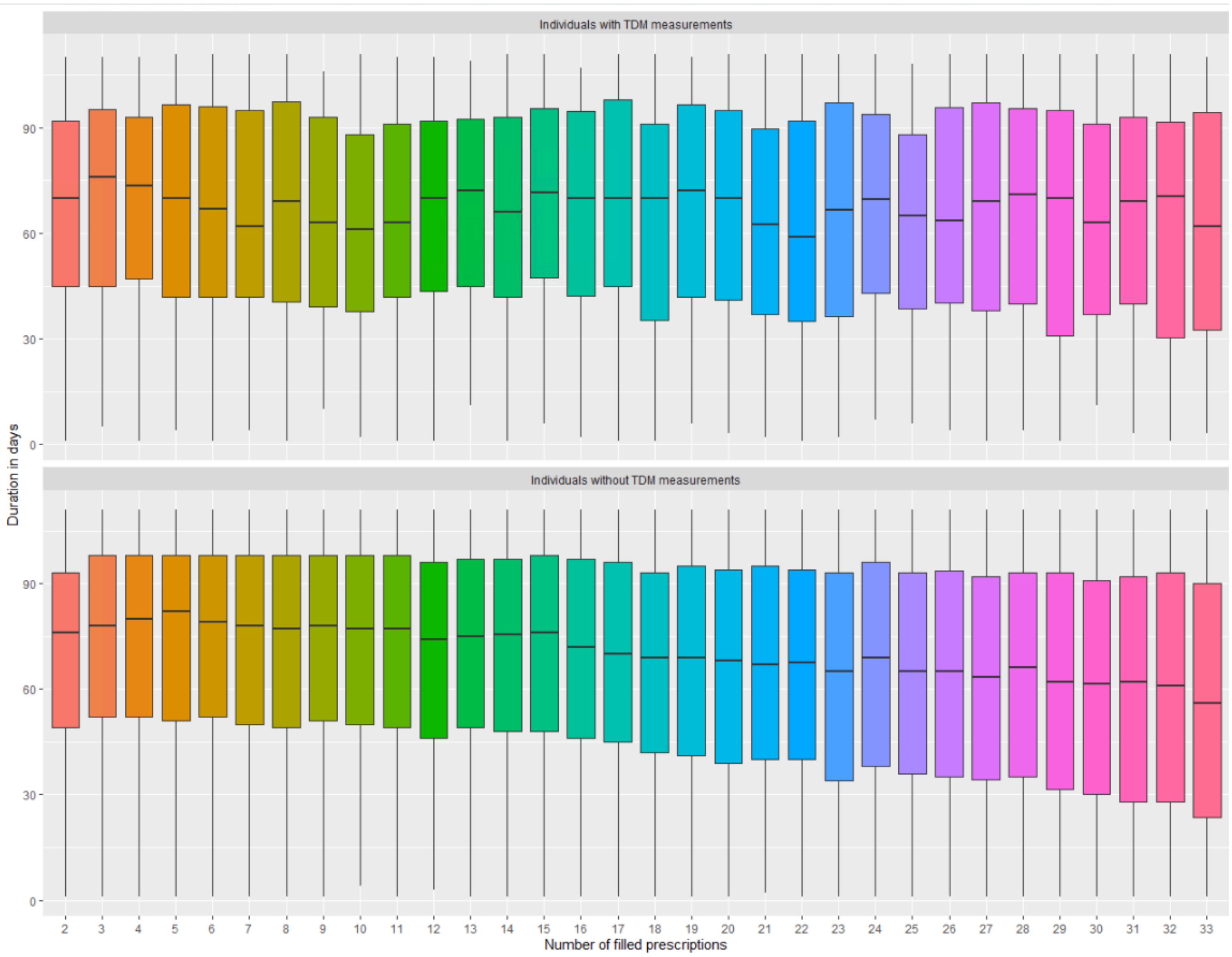

Supplementary figure 4. Median duration of the temporal distances between consecutive prescriptions for phenobarbital. *TDM = therapeutic drug monitoring*.

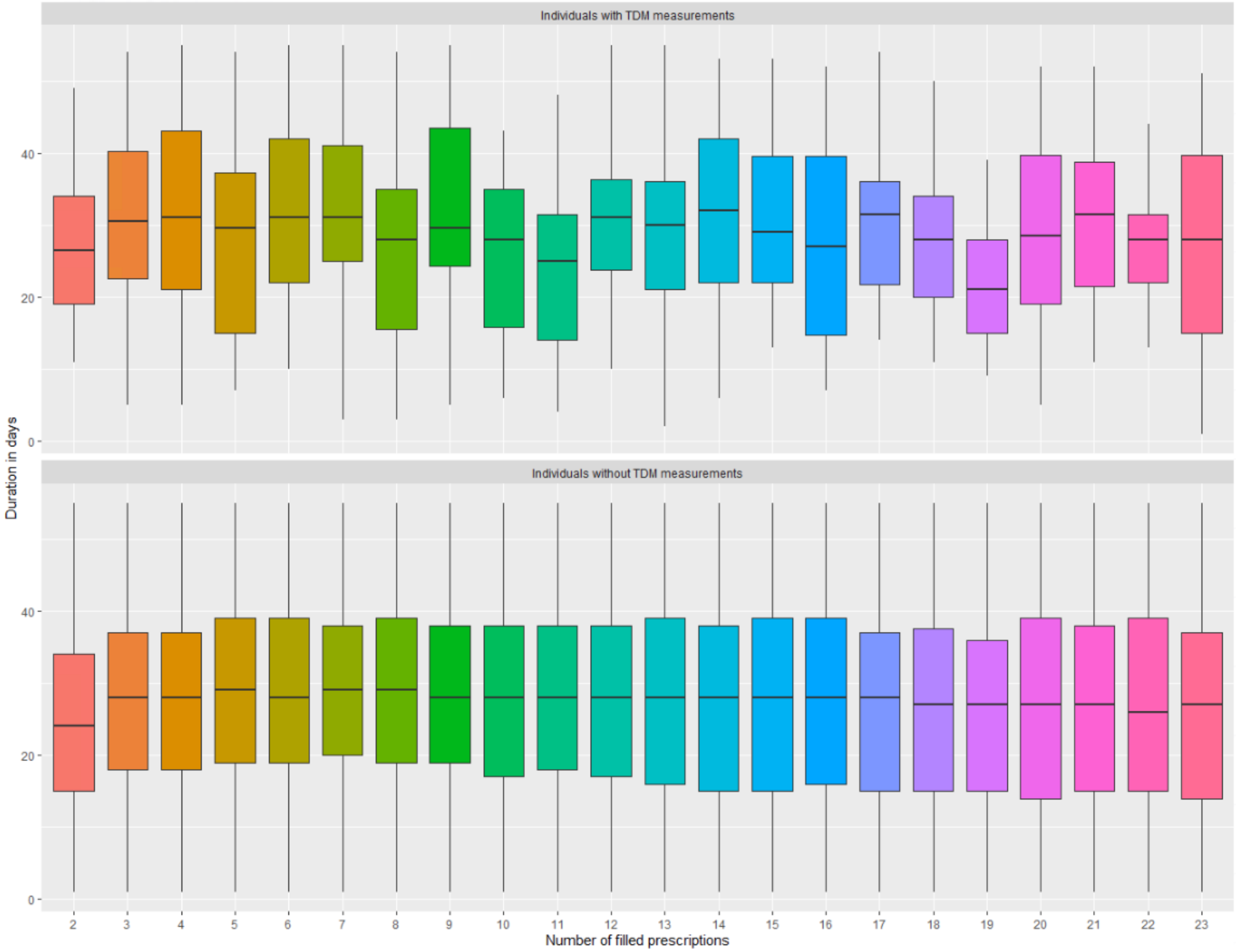

Supplementary figure 5. Median duration of the temporal distances between consecutive prescriptions for gabapentin. *TDM = therapeutic drug monitoring*.

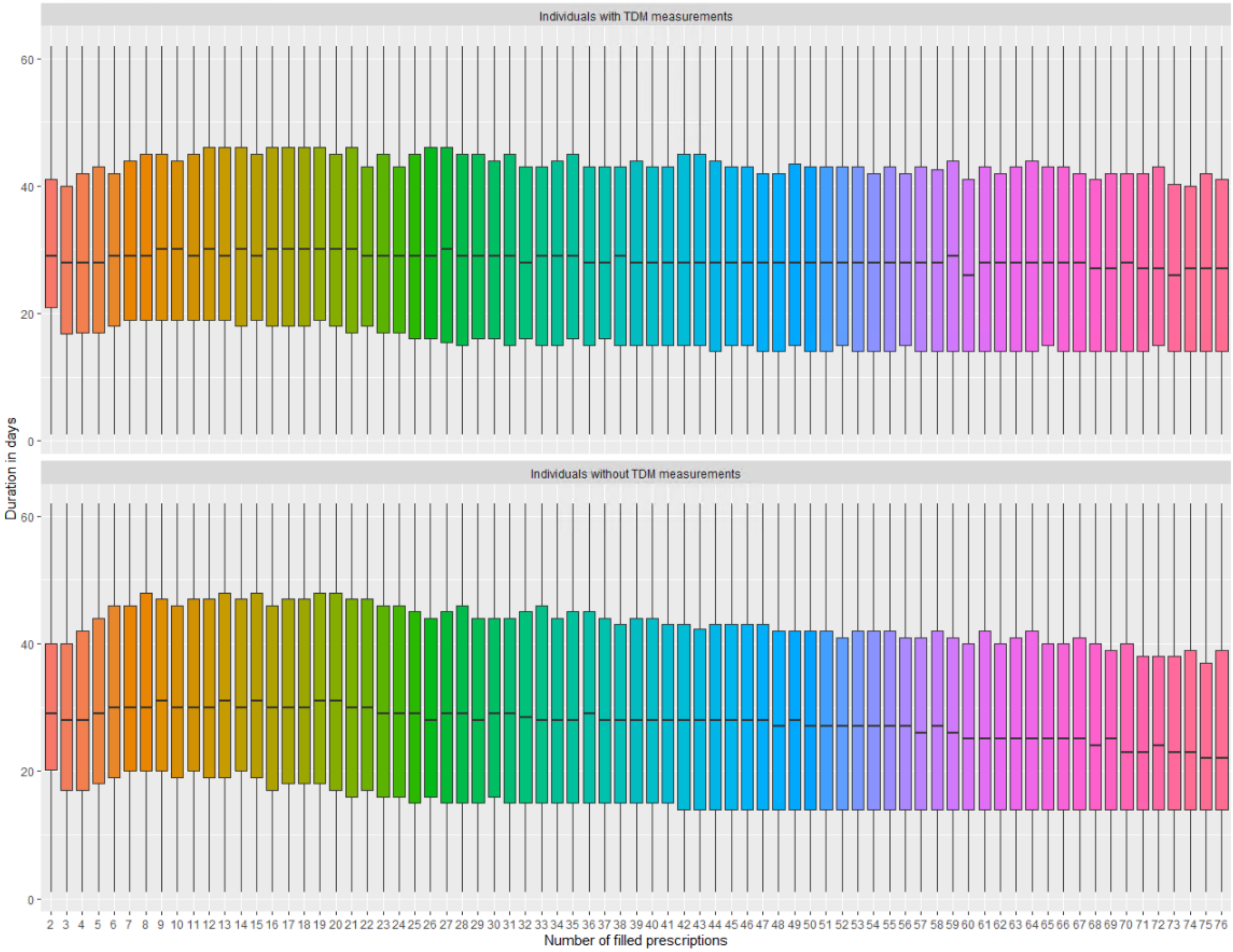

Supplementary figure 6. Median duration of the temporal distances between consecutive prescriptions for lamotrigine. *TDM = therapeutic drug monitoring*.

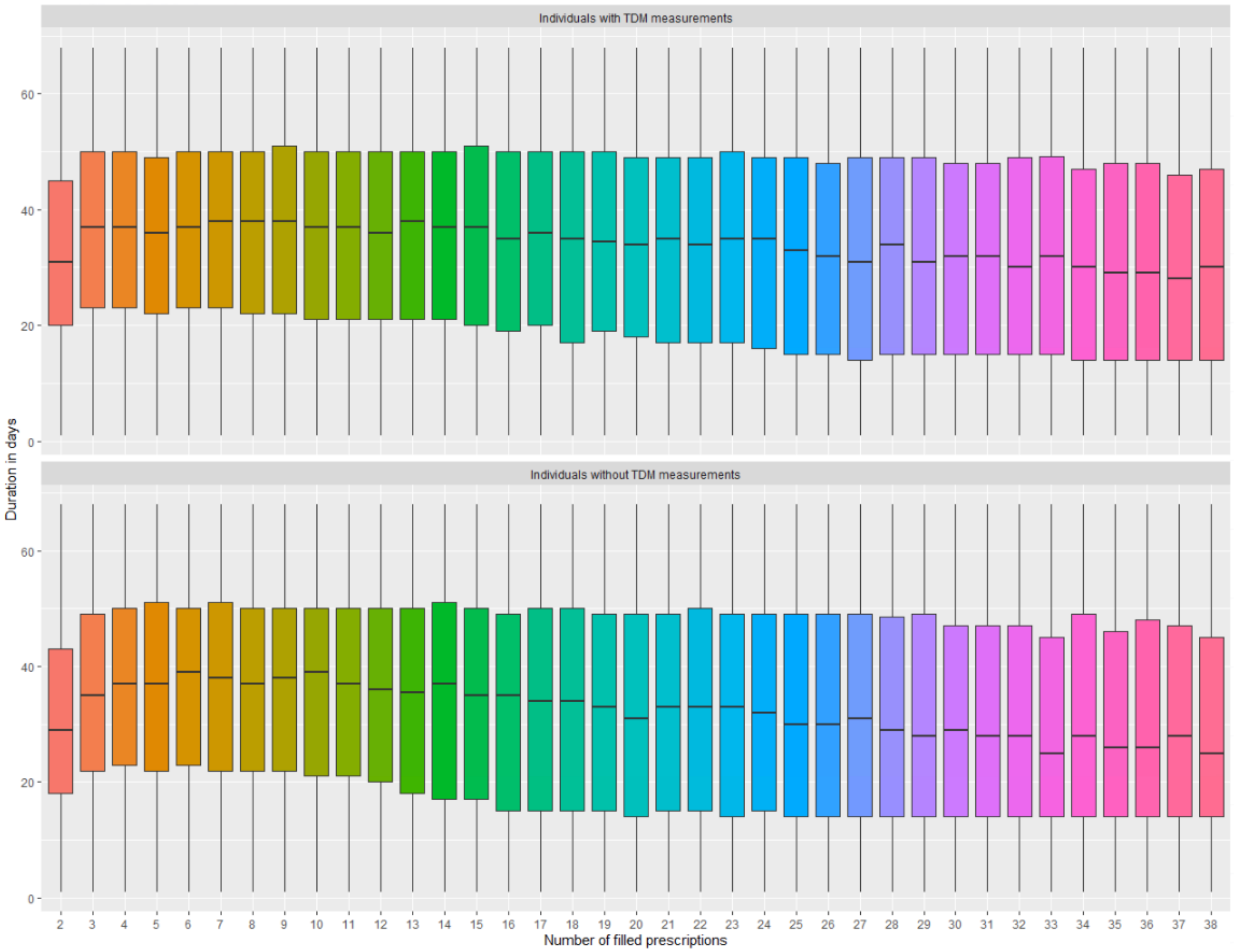

Supplementary figure 7. Median duration of the temporal distances between consecutive prescriptions for levetiracetam. *TDM = therapeutic drug monitoring*.

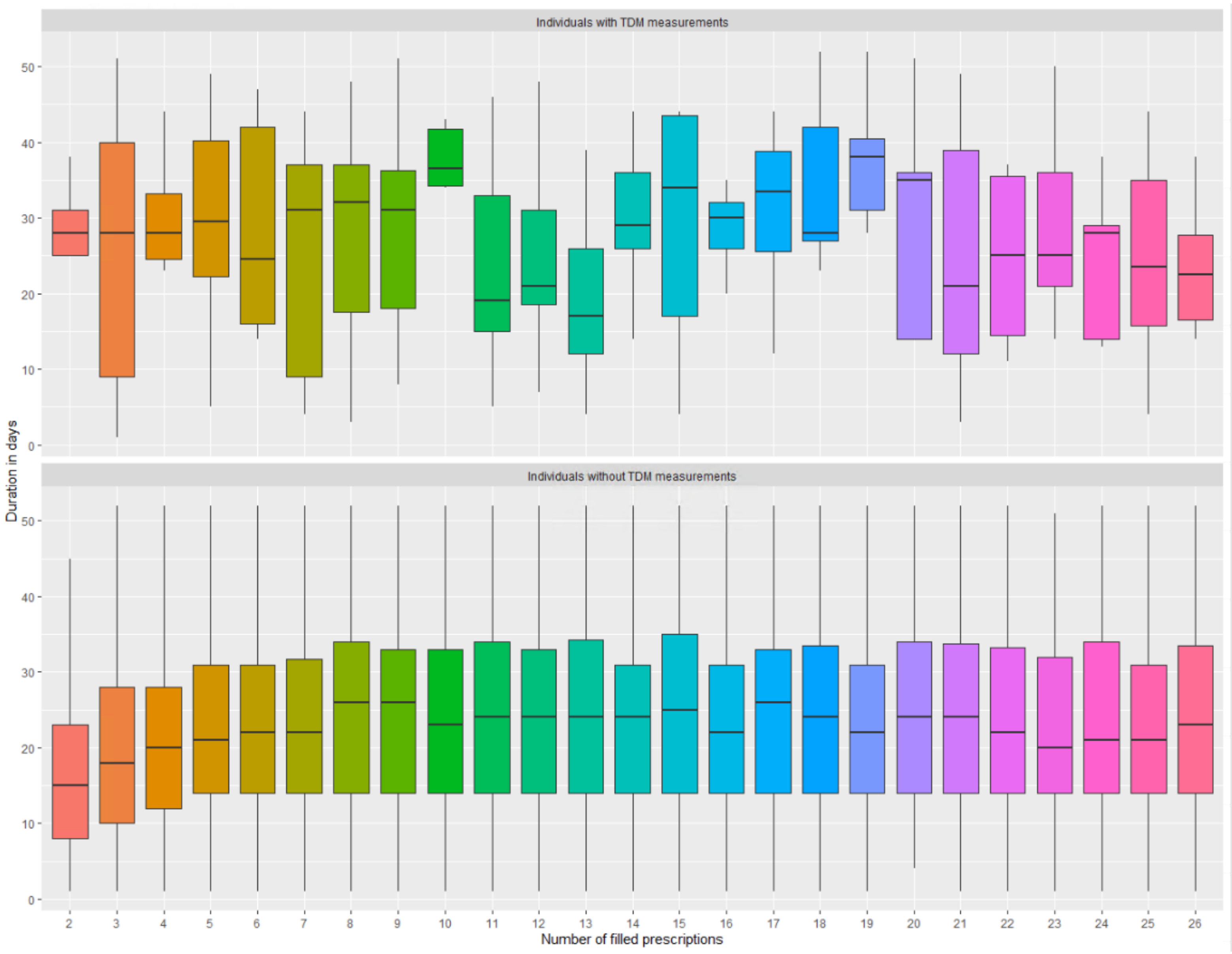

Supplementary figure 8. Median duration of the temporal distances between consecutive prescriptions for pregabalin. *TDM = therapeutic drug monitoring*.

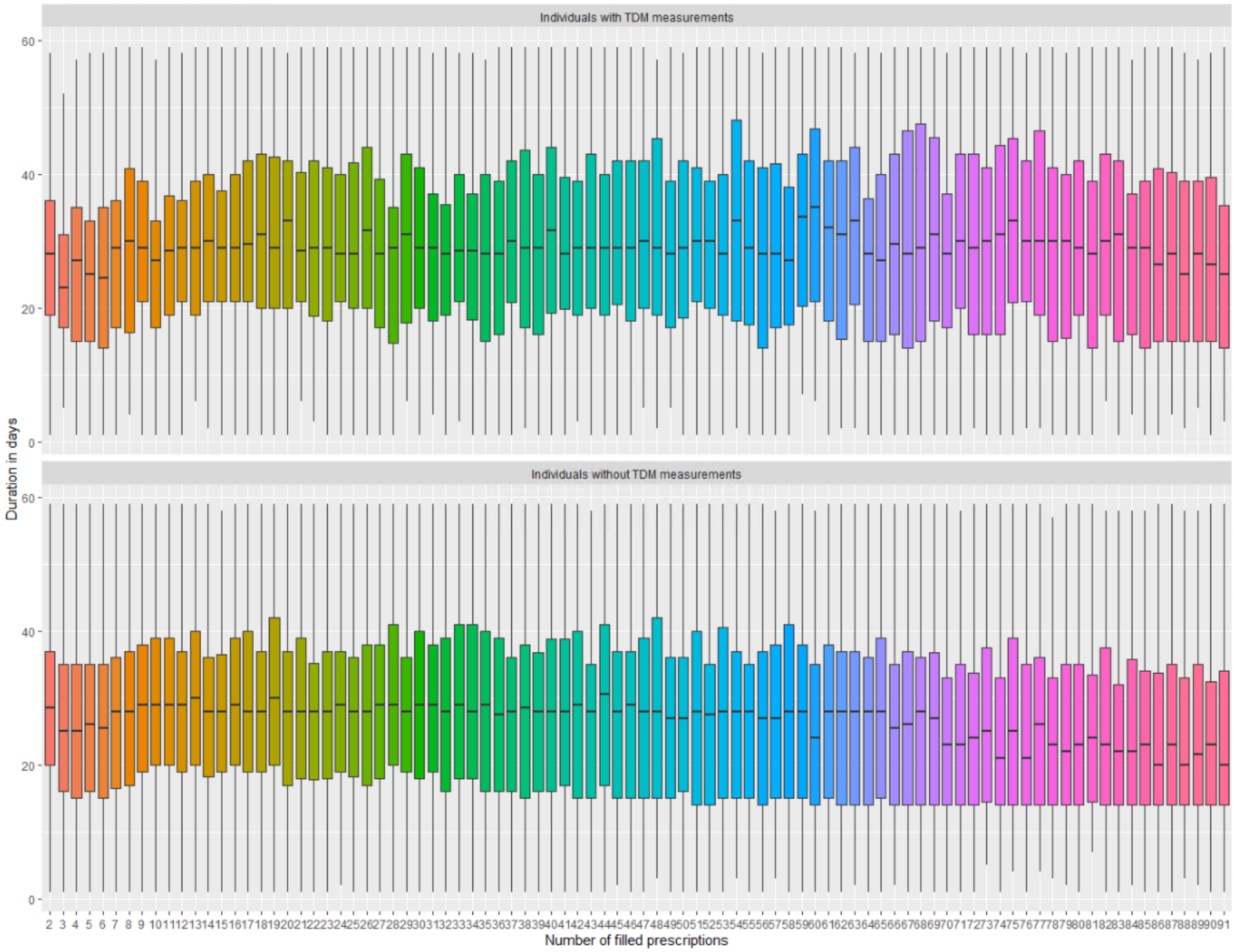

Supplementary figure 9. Median duration of the temporal distances between consecutive prescriptions for topiramate. *TDM = therapeutic drug monitoring*.

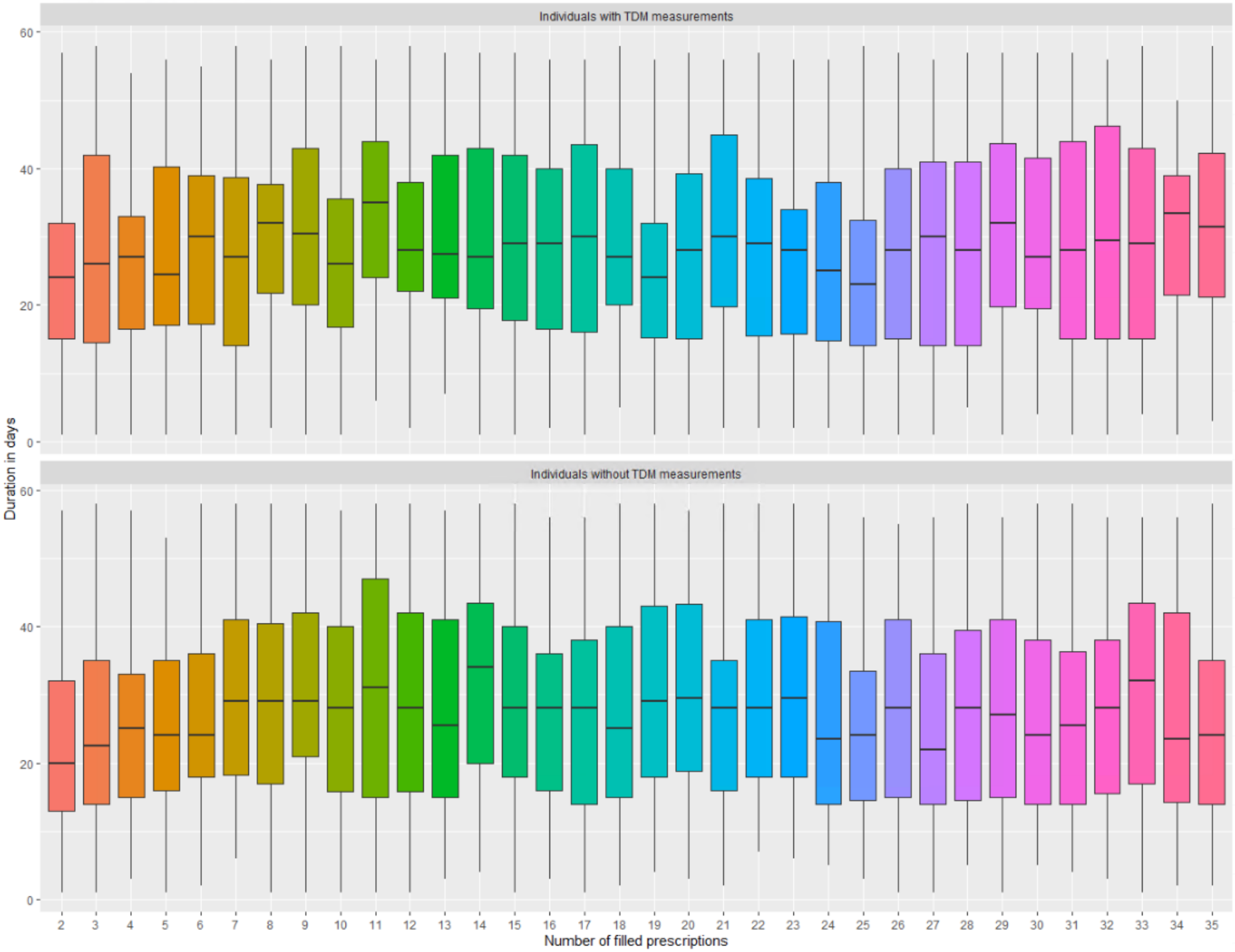

Supplementary figure 10. Median duration of the temporal distances between consecutive prescriptions for zonisamide. *TDM = therapeutic drug monitoring*.

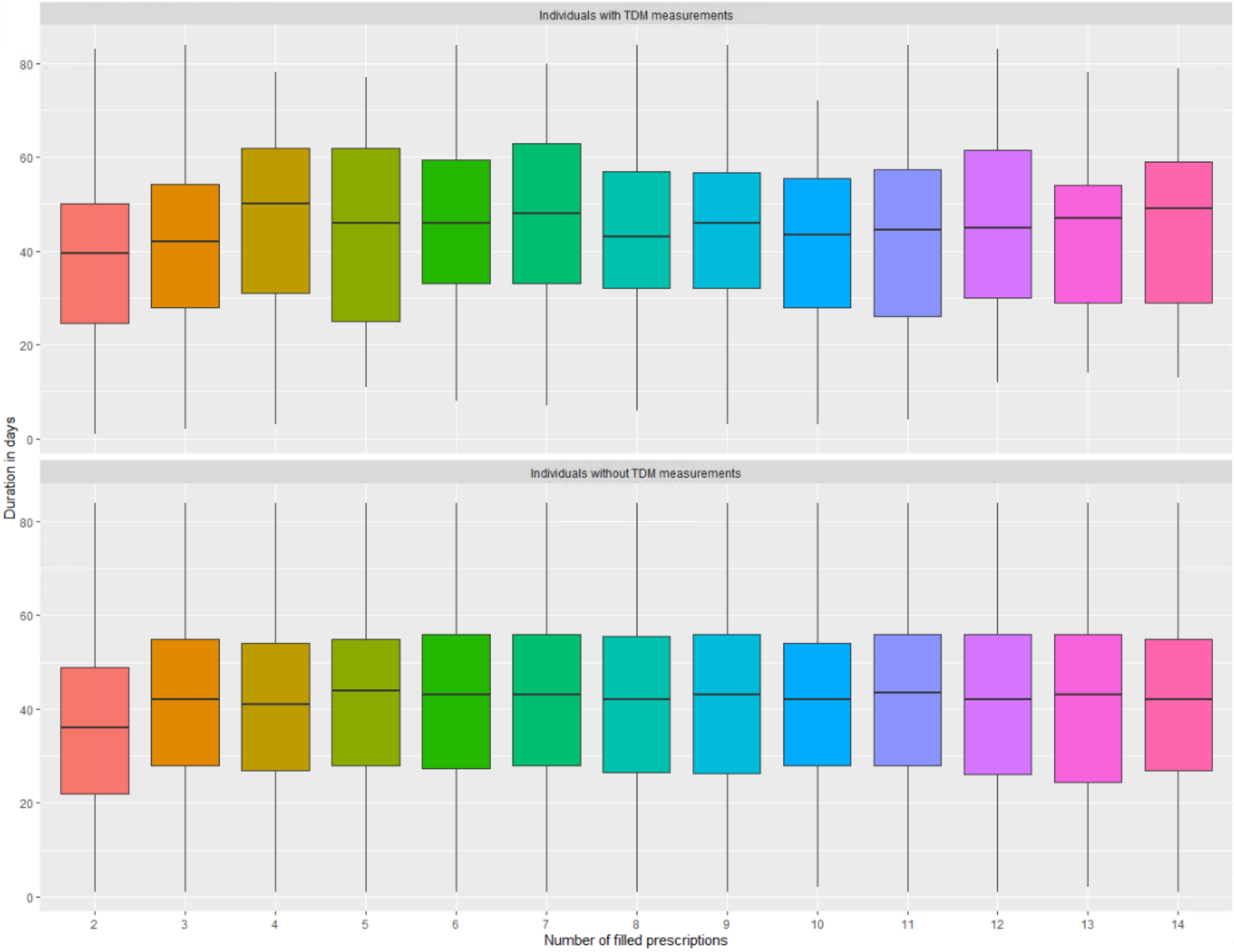

Supplementary figure 11. Median duration of the temporal distances between consecutive prescriptions for clobazam. *TDM = therapeutic drug monitoring*.

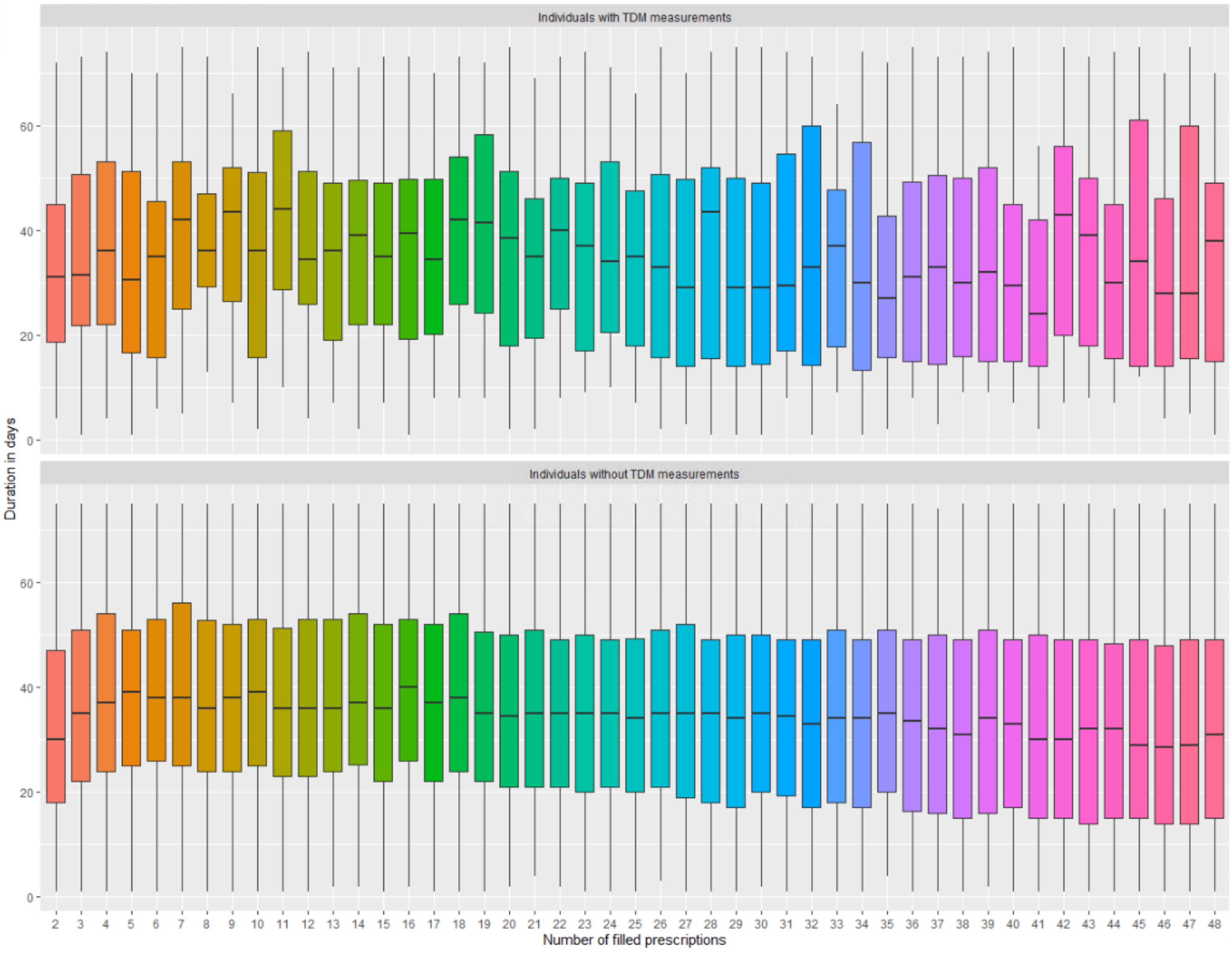

Supplementary figure 12. Median duration of the temporal distances between consecutive prescriptions for clonazepam. *TDM = therapeutic drug monitoring*.

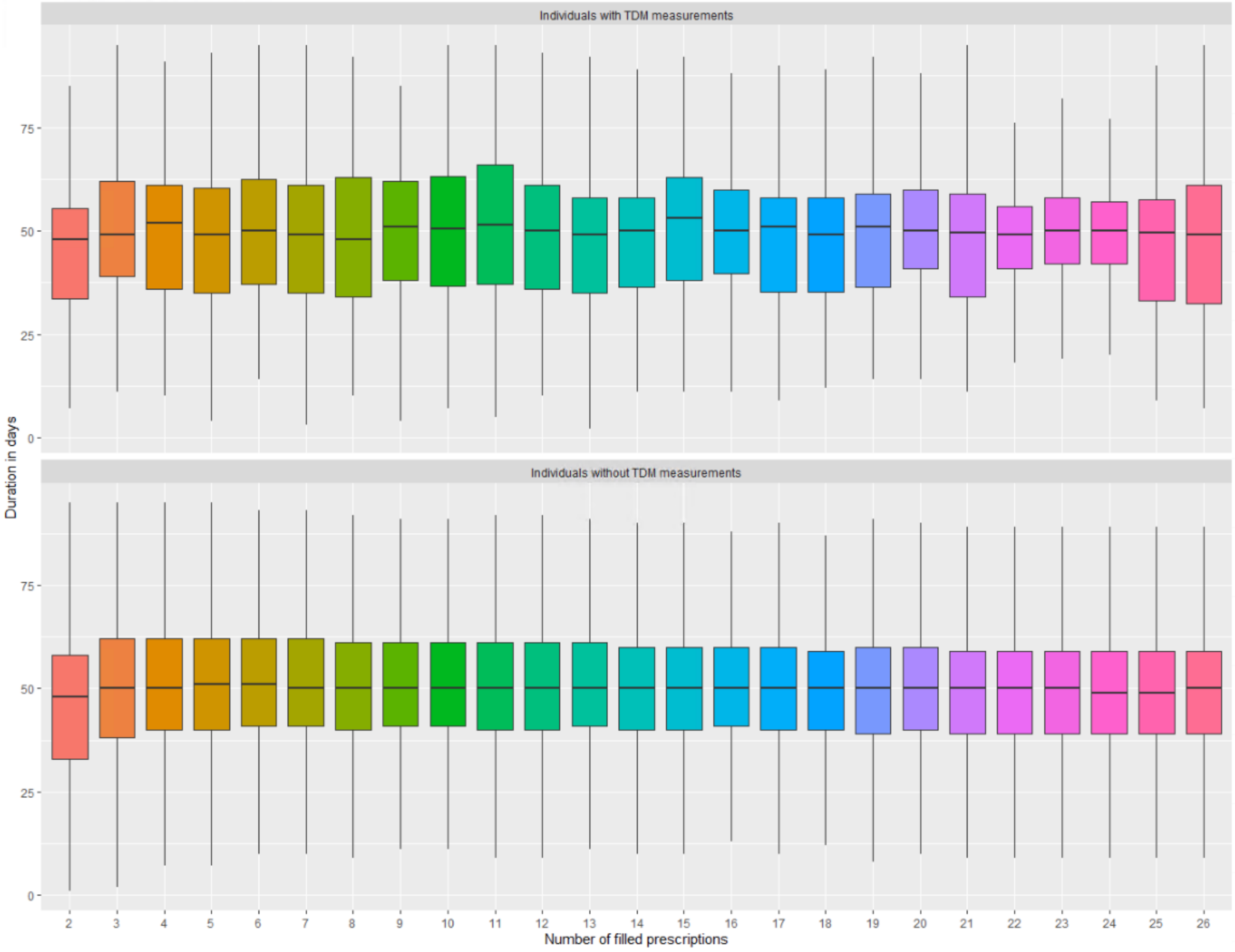

Supplementary figure 13. Median duration of the temporal distances between consecutive prescriptions for theophylline. *TDM = therapeutic drug monitoring*.

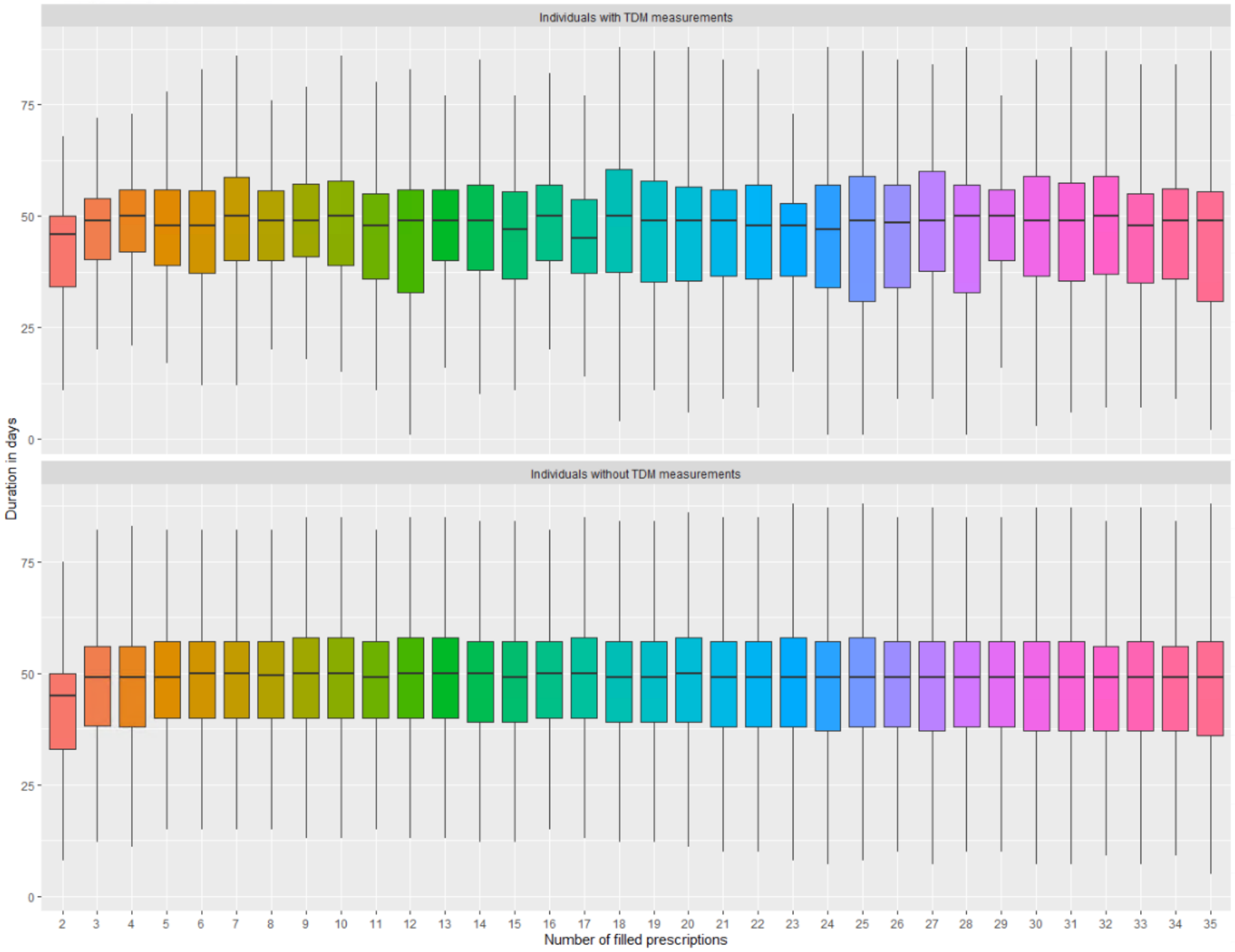

Supplementary figure 14. Median duration of the temporal distances between consecutive prescriptions for flecainide. *TDM = therapeutic drug monitoring*.

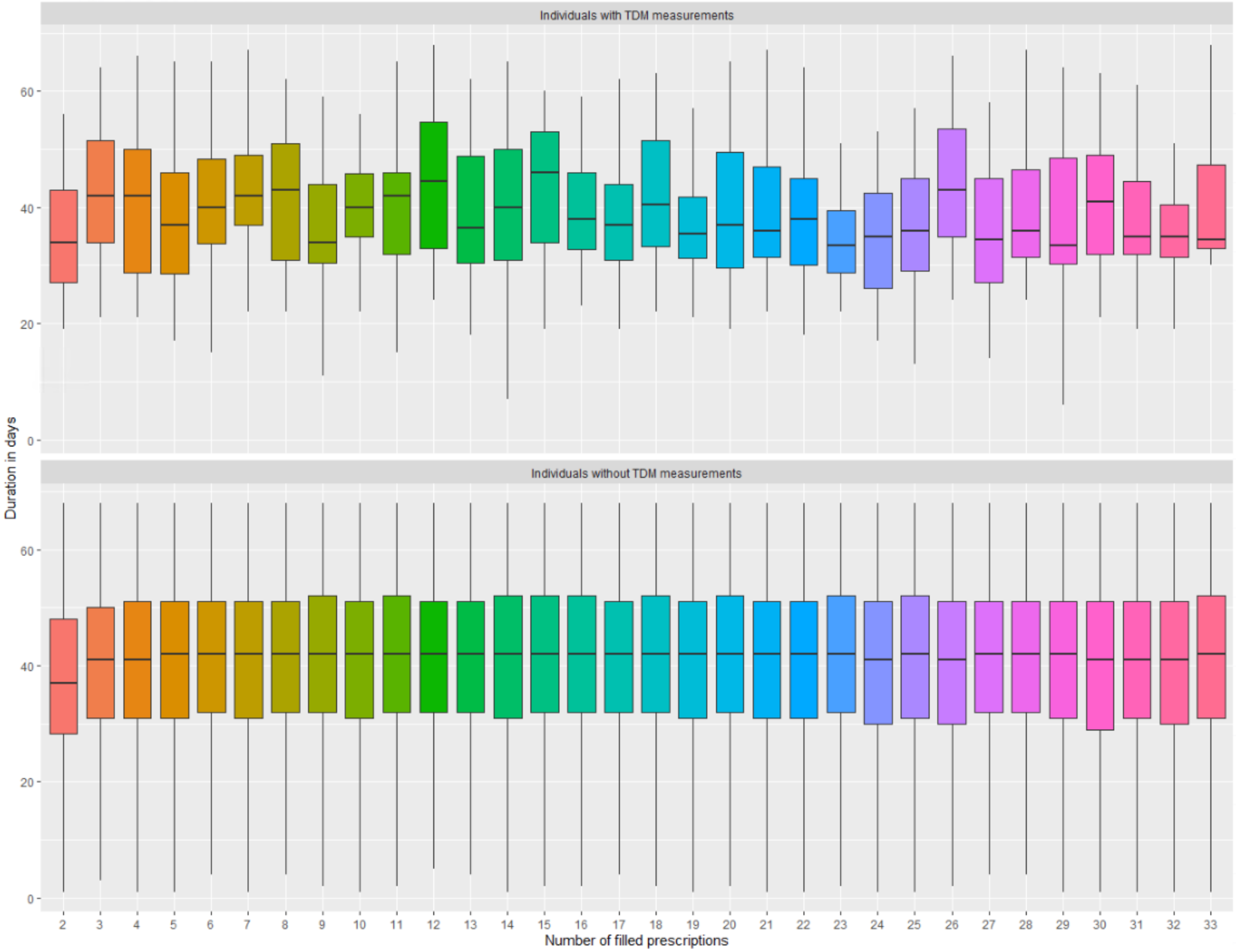

Supplementary figure 15. Median duration of the temporal distances between consecutive prescriptions for propafenone. *TDM = therapeutic drug monitoring*.

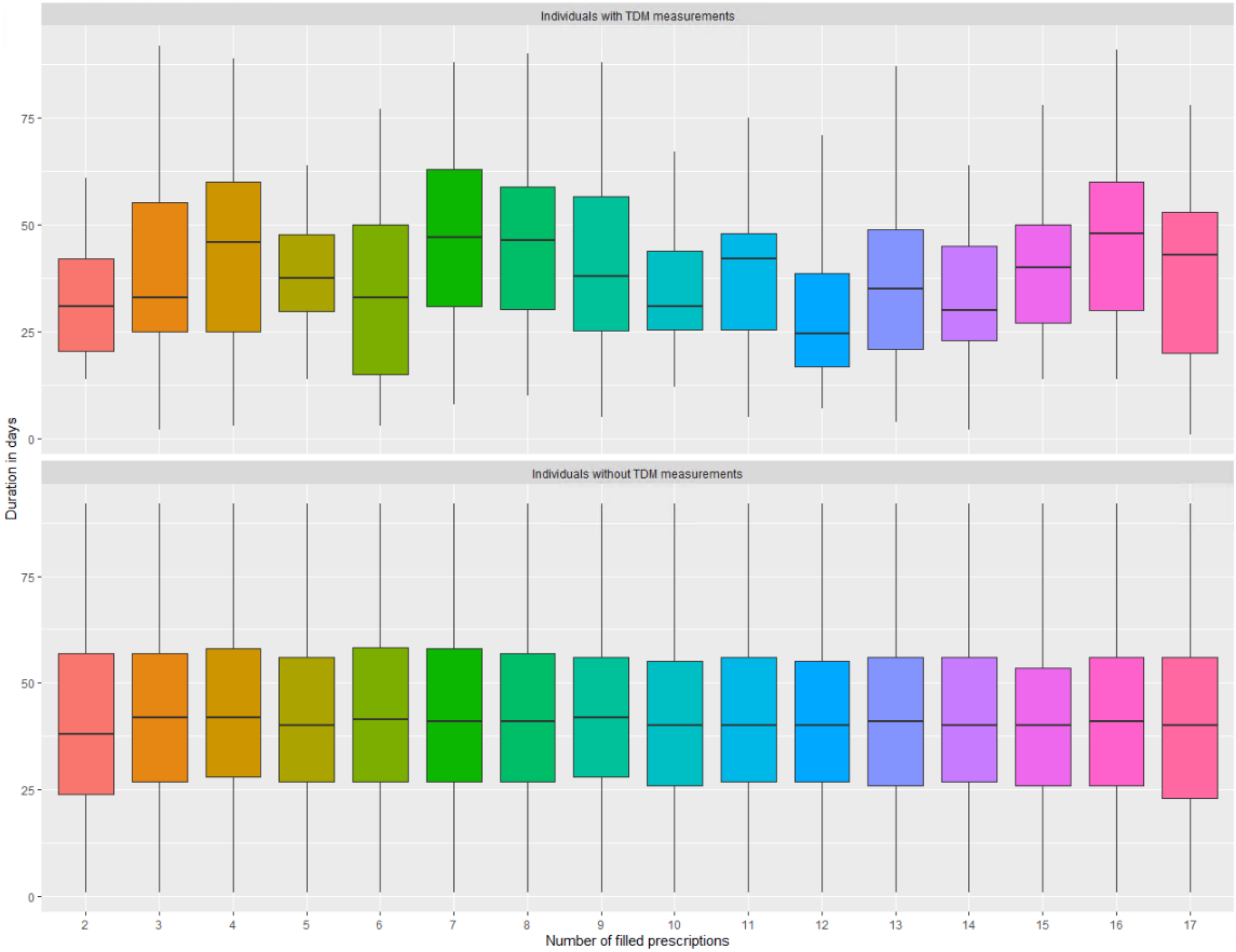

Supplementary figure 16. Median duration of the temporal distances between consecutive prescriptions for amitriptyline. *TDM = therapeutic drug monitoring*.

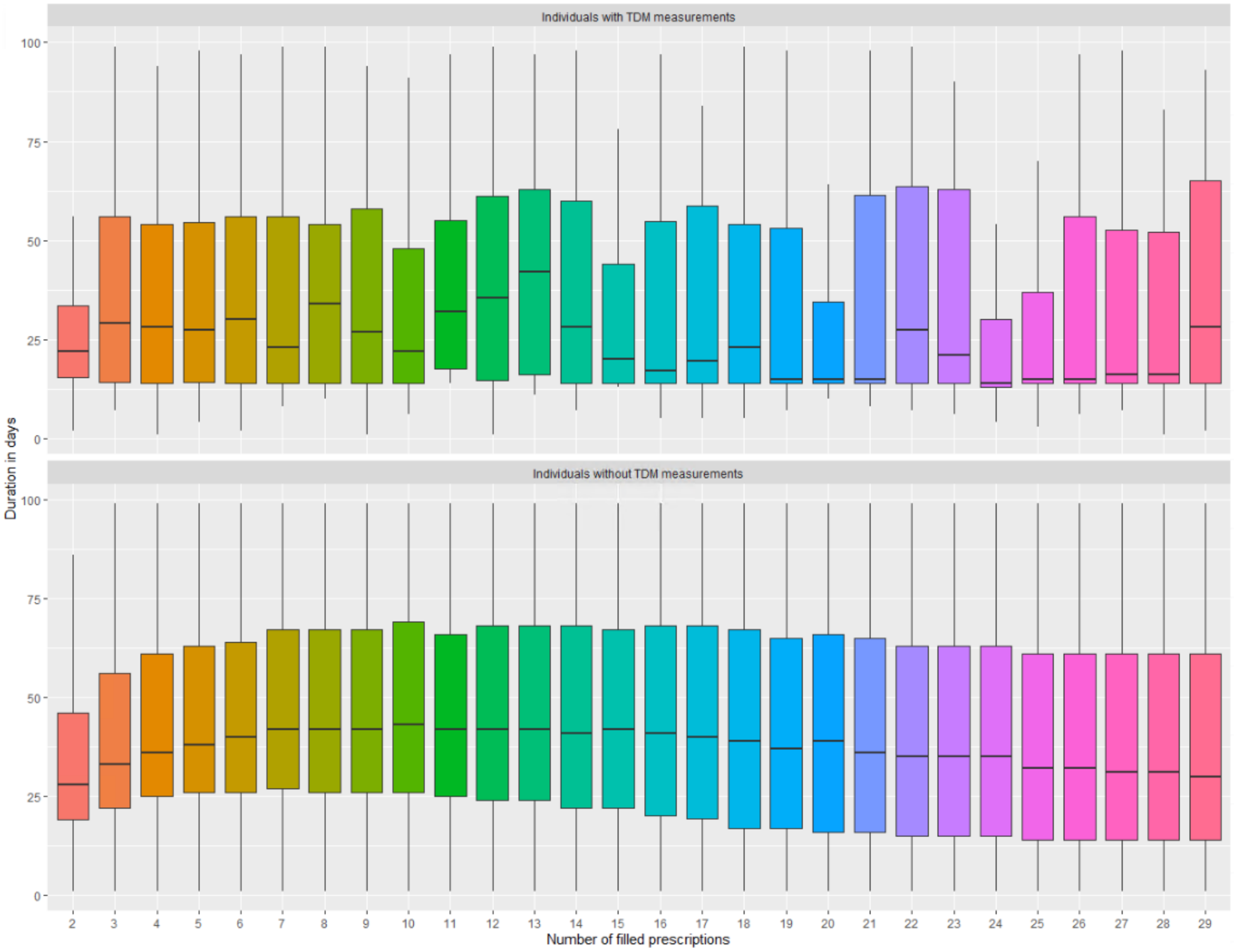

Supplementary figure 17. Median duration of the temporal distances between consecutive prescriptions for citalopram. *TDM = therapeutic drug monitoring*.

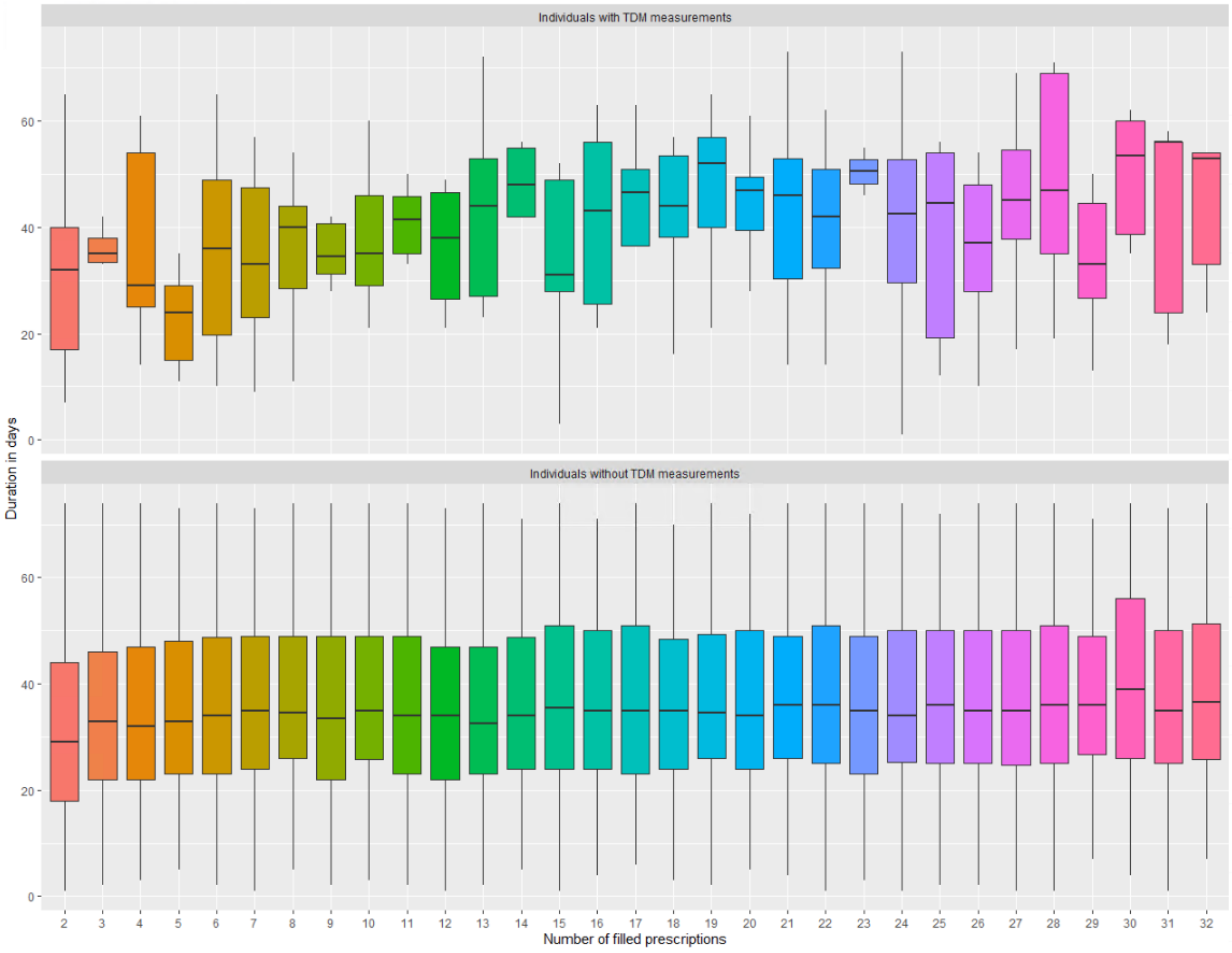

Supplementary figure 18. Median duration of the temporal distances between consecutive prescriptions for clomipramine. *TDM = therapeutic drug monitoring*.

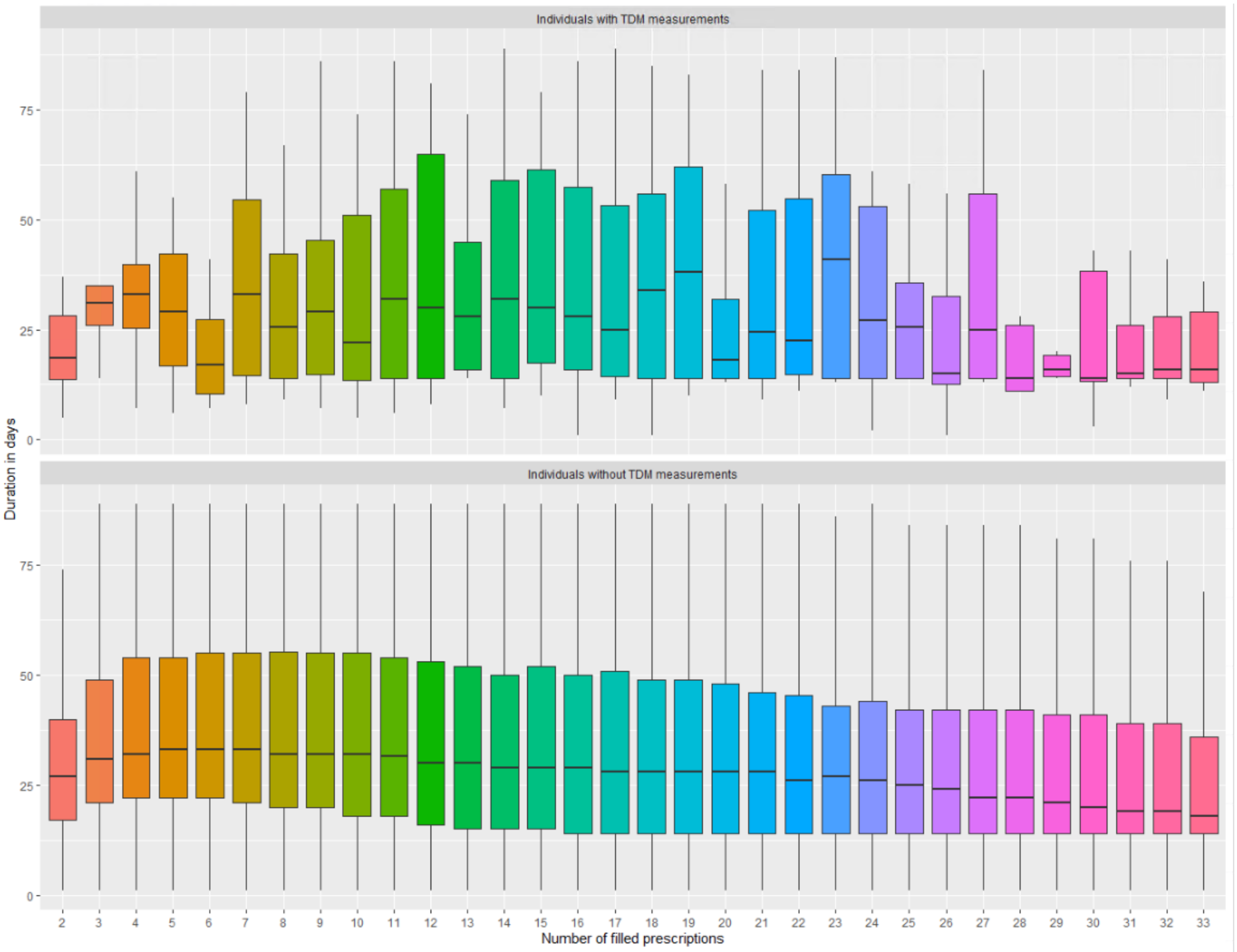

Supplementary figure 19. Median duration of the temporal distances between consecutive prescriptions for mirtazapine. *TDM = therapeutic drug monitoring*.

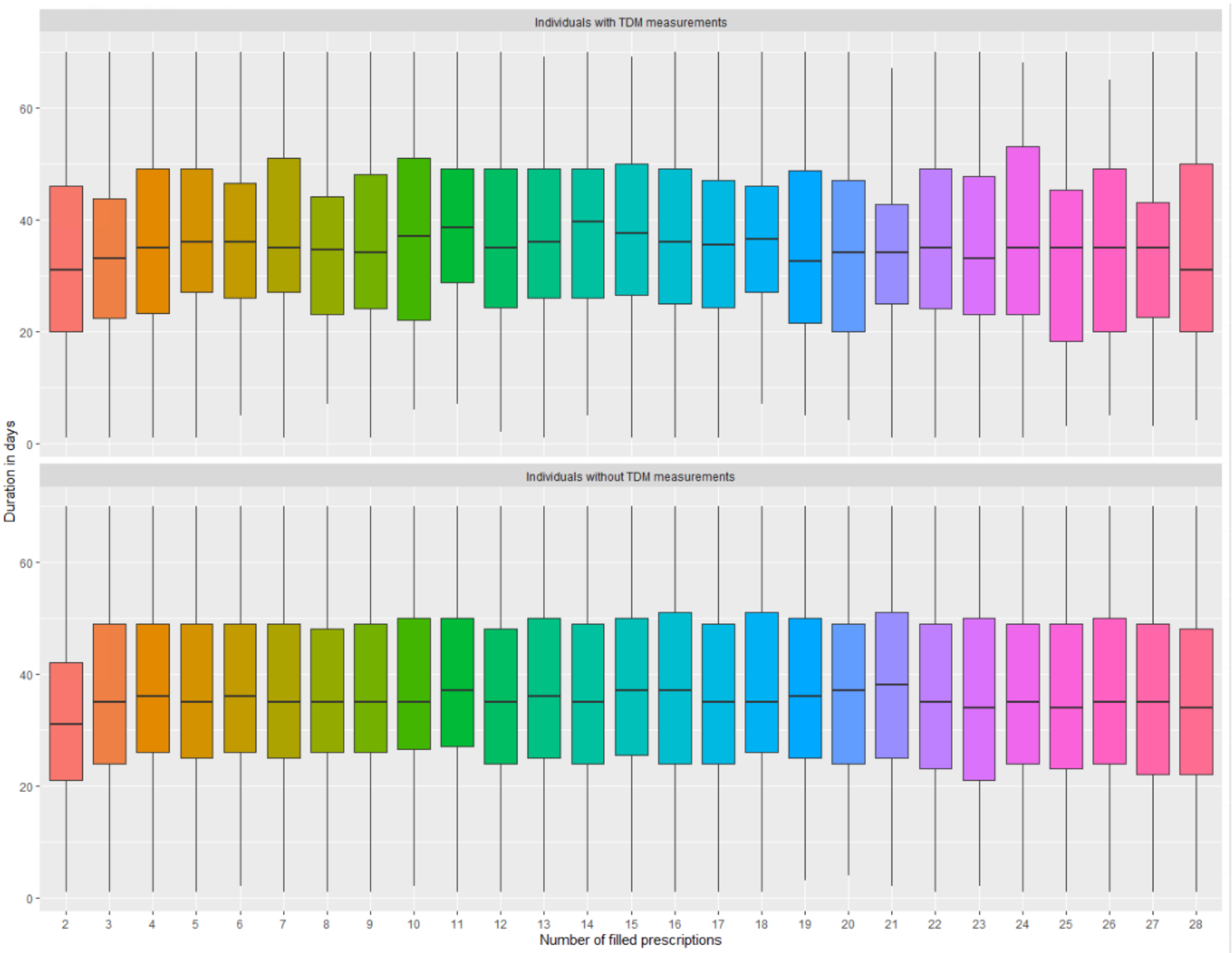

Supplementary figure 20. Median duration of the temporal distances between consecutive prescriptions for nortriptyline. *TDM = therapeutic drug monitoring*.
